# Specific ion effects directed noble metal aerogels: Versatile manipulation for electrocatalysis and beyond

**DOI:** 10.1126/sciadv.aaw4590

**Published:** 2019-05-24

**Authors:** Ran Du, Yue Hu, René Hübner, Jan-Ole Joswig, Xuelin Fan, Kristian Schneider, Alexander Eychmüller

**Affiliations:** 1 Physical Chemistry, Technische Universität Dresden, Bergstr. 66b, 01062 Dresden, Germany.; 2 College of Chemistry and Materials Engineering, Wenzhou University, Wenzhou 325000, China.; 3 Helmholtz-Zentrum Dresden-Rossendorf, Institute of Ion Beam Physics and Materials Research, Bautzner Landstrasse 400, 01328 Dresden, Germany.; 4 Theoretische Chemie, Fakultät für Chemie und Lebensmittelchemie, Technische Universität Dresden, 01062 Dresden, Germany.

## Abstract

Noble metal foams (NMFs) are a new class of functional materials featuring properties of both noble metals and monolithic porous materials, providing impressive prospects in diverse fields. Among reported synthetic methods, the sol-gel approach manifests overwhelming advantages for versatile synthesis of nanostructured NMFs (i.e., noble metal aerogels) under mild conditions. However, limited gelation methods and elusive formation mechanisms retard structure/composition manipulation, hampering on-demand design for practical applications. Here, highly tunable NMFs are fabricated by activating specific ion effects, enabling various single/alloy aerogels with adjustable composition (Au, Ag, Pd, and Pt), ligament sizes (3.1 to 142.0 nm), and special morphologies. Their superior performance in programmable self-propulsion devices and electrocatalytic alcohol oxidation is also demonstrated. This study provides a conceptually new approach to fabricate and manipulate NMFs and an overall framework for understanding the gelation mechanism, paving the way for on-target design of NMFs and investigating structure-performance relationships for versatile applications.

## Teaser

Specific ion effects are demonstrated to create and flexibly manipulate noble metal aerogels for versatile applications.

## INTRODUCTION

Investigation of functional porous materials (FPMs) is an everlasting topic standing at the cutting edge of materials science because the combined porous structures and versatile compositions of FPMs warrant their remarkable performance in widespread fields (*1*). Foams, which are equal to aerogels in certain cases, are one type of widely studied FPMs. They feature macroscopically monolithic structures favorable for handling and additional applications and can be structured from nearly any unit ranging from silica, nanocarbons, polymers, and two-dimensional (2D) materials to inorganic nanocrystals (*2*–*6*). As a rising star in the foam family, noble metal foams (NMFs) raised tremendous interest upon their debut (*7*–*9*). Imparting 3D gel networks with features of noble metals (e.g., high catalytic activity, high electrical conductivity, and plasmonic properties) has endowed NMFs with wide application potentials. However, their developments are still at the infant stage, where the fabrication strategies are more or less limited and structures/properties cannot be well manipulated.

NMFs are typically fabricated by four classes of methods, i.e., dealloying (*9*), templating (*10*, *11*), direct freeze-drying (*12*, *13*), and sol-gel process (*14*). Since only the sol-gel method has obtained great success in producing nanostructured and high–surface area NMFs under mild conditions, it immediately became the most popular synthetic strategy upon its introduction (*14*–*19*). Because the sol-gel–derived foams are in good agreement with the definition for aerogels, the term noble metal aerogels (NMAs) will be used in this manuscript afterward. Conventionally, NMAs were prepared by ultracentrifugation and subsequent destabilization of the nanoparticles (NPs) solution to obtain noble metal hydrogels (NMHs) in 1 to 2 weeks, followed by supercritical drying to afford the corresponding aerogels (*14*). Afterward, considerable efforts were made to enhance gelation kinetics, modulate microstructure, and simplify fabrication procedures. Appropriate destabilizers (e.g., CaCl_2_) (*15*) or elevated temperature (333 K) (*16*) have been adopted to reduce the gelation time to several hours or several minutes depending on the precursor concentrations (0.3 to 10 mM). Using Ag nanoshells (*17*) or PdNi hollow spheres (*18*) as building blocks, hierarchically structured NMAs with unique optical/electrochemical properties were obtained. To avoid the considerable costs incurred by the concentration process, Liu *et al*. (*19*) substantially simplified the gelation to one step, directly initiating Pd/Pt gels using specific amounts of NaBH_4_.

Despite substantial progress, the sol-gel method for preparation of NMAs is still at its infant stage, and numerous mysteries remain in this process, which largely constrains the available systems, impedes the understanding of the gelation mechanism, and blocks on-demand manipulation. For example, regardless of the gelation method, ligament sizes usually fall in the range of 3 to 6 nm for Pd/Pt and >100 nm for Au (*14*–*16*, *19*, *20*). Many strategies work well with the Pd/Pt/alloy systems but fail in obtaining nanostructured gold gels (*14*–*16*, *19*). Appealing core-shell–structured NMAs are obtained only in the form of powders, not to mention the tedious procedures involved (*21*). Moreover, until now, the systematic modulation of the ligament size and corresponding physicochemical properties have not been realized. In addition, investigations of applications/properties of NMAs are almost only restricted to electrocatalysis. Therefore, from both fundamental and practical considerations, it is high time to develop distinct strategies that not only could fabricate and flexibly manipulate NMAs but also could serve as a platform to study the gelation mechanism.

Salting out is a long-known method to destabilize colloidal particles from solutions. This process is usually explained on the basis of the DLVO (Derjaguin-Landau-Vervey-Overbeek) theory, where introduced “undifferentiated” electrolyte ions raise the ionic strength, thus electrostatically screening charged particles and inducing aggregation. However, specific ion effects emerge with increasing concentration (e.g., ≥100 mM), and the Hofmeister series was proposed thereafter to arrange ions following their ability to salt out proteins (*22*–*24*). Thus, if the chemical diversity of different ions could be activated, unprecedented opportunities will be unlocked to engineering the gelation process and the corresponding gels. Although CaCl_2_ (≤1 mM) and NaCl (8 to 250 mM) have been used to prepare NMAs (*15*, *25*), the adopted concentration is not suitable for unlocking specific ion effects, and the systematic study of the specific ion effects is in absence. A handful of works found the role of specific anions in regulating the gelation behavior of supramolecular and polymer gels (*26*, *27*), while the role of cations was scarcely considered. In this study, we present the rapid fabrication and flexible manipulation of NMAs by activating and designing specific ion effects. By in-depth studying the gelation process experimentally and complementing DFT calculations, the specific ion–directed mechanism is proposed, and the overall reaction process is outlined. On this basis, versatile manipulations including compositions (Au, Ag, Pd, Pt, and alloys), ligament sizes (6.9 to 113.7 nm for gold and 3.1 to 142.0 nm for others), specific surface areas (2.5 to 29.7 m^2^ g^−1^ for gold and 3.4 to 122.7 m^2^ g^−1^ for others), and spatial element distribution (e.g., core-shell) are realized. The enormous ion library and the generality of the presented method will offer unprecedented opportunities for further manipulation of NMAs and extending to diverse colloidal solution systems. Last, the interesting compression-induced dark-to-shining transition phenomenon, the programmable self-propulsion behavior, and the remarkable activity in electrocatalytic alcohol oxidation of as-prepared NMAs are demonstrated, suggesting their huge potentials in extensive fields.

## RESULTS AND DISCUSSION

### Overall gelation process

**Fig. 1:**
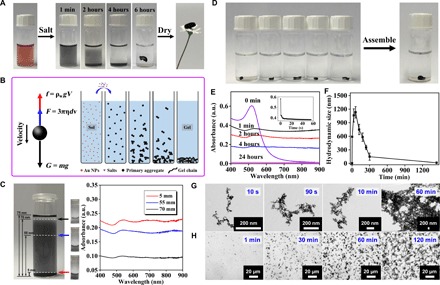
Analysis of the overall gelation process of gold NPs. (**A**) Digital photos of the gel preparation process. (**B**) Schematic demonstration of the gelation process and a corresponding force analysis. (**C**) The gradient distribution during the gelation characterized by ultraviolet-visible (UV-vis) absorption spectra. a.u., arbitrary units. (**D**) Several pieces of as-prepared hydrogels can assemble to one piece. (**E** to **H**) Time-lapse (E) UV-vis absorption spectra, (F) hydrodynamic size, (G) transmission electron microscopy (TEM), and (H) optical microscopy characterization during gelation. The inset in (E) shows the time-lapse UV-vis absorption evolution at 510 nm, which was recorded during the first minute upon reaction. (Photo credit: Ran Du.)

Briefly, we added the as-prepared gold NP solution with specific salts and grounded it 4 to 12 hours to yield the hydrogel, which was further freeze-dried to obtain the corresponding aerogel (Fig. 1A, fig. S1, and movie S1). Here, the concentration of the metal precursors is denoted as *c*_M_. Notably, the gel forms across wide *c*_M_ (0.02 to 2 mM). The lowest *c*_M_ (0.02 mM, ~5 days for gelation) was one to four order of magnitude lower than that of all reported NMAs to date (0.2 mM to above 100 mM) (*14*–*21*, *25*), indicating a robust gelation ability and completely eliminating needs for expensive concentration processes. In contrast, it is impossible to destabilize the gold NP solution (0.2 mM) by frequently used methods (*14*–*16*, *18*) even with prolonged time (e.g., 3 days), such as addition of oxidants or adopting elevated temperature (e.g., 348 K). The long gelation time is a common issue for preparing NMHs. Although it has been cut down to several minutes or several hours (*15*, *16*), the expensive concentration process or the elevated temperature involved can incur high costs or unfavorable structures. Hence, our approach displays unique advantages for rapid gelation at simultaneously low *c*_M_ and ambient temperature.

More formidable issues remain in regulating multiscale structures and properties of NMAs. These challenges are largely attributed to the ambiguous understanding of the gelation process, which has only rarely been studied previously. To address these issues, we thoroughly study the gelation process, which is pivotal in determining the as-obtained NMAs. As illustrated in Fig. 1A and fig. S1, the red gold NP solution immediately turned black upon reaction and displayed a vertical color gradient afterward, lastly forming an extremely flexible hydrogel film at the bottom. This phenomenon is in sharp contrast with most systems of other materials (*2*, *4*, *5*, *28*), where only the concentrated solution can afford free-standing gels, which are of similar size as the original volume. To explain this unconventional phenomenon, we propose a gravity-driven assembly model. As seen from Fig. 1B, salt-initiated aggregates gradually grow and settle down driven by gravity (see the Supplementary Materials) and lastly concentrate and evolve into the hydrogel at the bottom. To support the above model, we recorded ultraviolet-visible (UV-vis) absorption spectra of a “halfway” gel system at different heights (Fig. 1C), unambiguously manifesting a gradient distribution along the gravity direction. In addition, we demonstrate that aggregates are active during the entire gelation process, which is evident from the spontaneous assembly of several fresh hydrogels into one monolithic gel (Fig. 1D). Hydrogels can repair themselves after destruction (fig. S2), displaying promising self-healing properties in diverse environments without external energy input. This remarkable self-healing behavior may account for the observed monolithic structure of NMHs despite their fragility.

We further reveal the holistic picture of the gelation process by performing several time-lapse characterizations. In situ UV-vis absorption spectra show an instant change from the characteristic surface plasmon resonance (SPR) absorption of gold NPs (~514 nm) to broadband absorption upon reaction (Fig. 1E). We performed the single-wavelength test within the first minute upon reaction as illustrated in the inset of Fig. 1E. It shows that the SPR absorption of gold NPs disappeared within seconds, characterizing extremely fast formation of aggregates with multiscale microstructures upon addition of the salts (*29*). The following intensity decrease agrees with the proposed gravity-driven sedimentation process. The in situ dynamic light scattering (DLS) measurement indicated that the hydrodynamic size (*d*_h_) of NPs/aggregates rapidly increases from 5.0 ± 0.2 to 589 ± 11 nm within 2 min and achieves a maximum of 1131 ± 126 nm at ~60 min (Fig. 1F). Because of a positive correlation between the sedimentation speed and the particle size (see the Supplementary Materials), larger aggregates fall down quickly and leave smaller ones in the solution phase, resulting in decreasing aggregate sizes with prolonged time. In this way, we obtained a volcano-shaped *d*_h_-time curve due to the competition between growth and sedimentation of aggregates. Time-lapse transmission electron microscopy (TEM) and in situ optical tests further reveal the evolution footprints of 3D networks directly at different scales (Fig. 1, G and H). The anisotropic growth of 0D NPs to 1D nanowires can be attributed to the anisotropic character of electrostatic repulsion. After the formation of gold NP dimers, the additional NPs will preferentially attach to the dimers from their ends rather than from their sides due to less energy costs (*30*). By repeating this process, NPs will grow mainly along the axial directions and eventually form nanowire-structured networks. Thus far, we present an overall picture of the sol-gel process by combining multiscale imaging techniques (10^−9^ to10^−1^ m), spectra analysis, and light scattering measurements.

### Specific ion effects in gelation

**Fig. 2:**
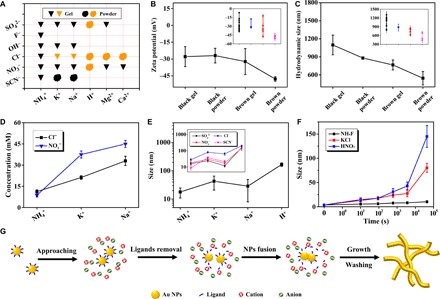
Analysis of the specific ion effects on the gelation behavior and ligament size. (**A**) Summary of the status of gels induced by different ions. The inverted triangle and the diffused circle indicated the gel and powder, and black and brown indicated the color of the products. (**B**) Zeta potential upon reaction and (**C**) *d*_h_ versus the color and form of products. The data were obtained by averaging detailed values from the inset diagram. (**D**) The low-threshold gelation concentration of salts (*c*_s_) versus the used cations. (**E**) The ligament size (averaged over the anions used as in the inset diagram) of as-synthesized gold aggregates versus cations. (**F**) Time-lapse ligament size evolution of gold aggregates induced by three typical salts. (**G**) Proposed mechanism for gel formation.

To manipulate NMAs, the microscale mechanism accounting for the NP growth needs to be studied in depth. In the present system, gelation was induced by high-concentration salts (~10^2^ mM) compared to previous reports (*15*), satisfying the requirements to activate specific ion effects dictated by the Hofmeister series (*23*). Therefore, the current system may not only serve as an excellent platform to study the specific ion effects in fabricating noble metal gels but also provide an efficient tool to engineer the structures and the corresponding properties of NMAs. Here, 24 salts were selected and arranged according to the Hofmeister series, where the salting-out effect decreased from SO_4_^2−^ to SCN^−^ for anions and from NH_4_^+^ to Ca^2+^ for cations (table S1) (*22*, *24*). As summarized in Fig. 2A, different ions imposed prominent effects on the color and the form of the final products (fig. S3 and S4), implicating successful activation of specific ion effects. The change of the color suggests the variation of the characteristic size of gold, while the difference in forms (powders or gels) implicates the disparate interactions of the building blocks inside the products. These changes unambiguously indicate that the features of specific ions have been activated, enabling the modulation of the reaction pathways and thus the eventual structures. Generally, products changed from black to brown, from gels to powders induced by salts from the top left to the bottom right, roughly obeying the Hofmeister series. This trend is more evident for cations (along the *x* axis), which might be due to the enhanced cation-NP interaction resulting from small cation-NP distances, which are induced by electrostatic attraction. The partially inversed order for double-charged Mg^2+^ and Ca^2+^ is presumably attributed to quite different surface charge environments of different valance ions, which is common for the anomalous Hofmeister series presented in specific cases (*24*). In contrast, anions may display complicated effects, involving both salting-out effect and competitive adsorption with citrates, thus leading to the gelation behavior that deviates from the Hofmeister series (*7*). It is unexpected to find that Cl^−^ played an unusual role in boosting the ligament sizes, while the underlying mechanism is still under investigation.

On the basis of the observed phenomenon, the question arises how ions affect the color and form of the products. As dictated by the Hofmeister series, gold NPs should be salted out less effectively following the decreasing salting-out ability from NH_4_^+^ to H^+^, presumably leading to gradually incomplete 3D networks, i.e., a gel-to-powder transition. On the other hand, the color could reflect the ligament size to some extent. The black color often suggests small and hierarchical microstructures resulting from multiple absorption and scattering of light between numerous small-sized grains, while increasing ligament sizes will direct the color of the materials to that of bulk gold (*29*). The reason behind the ligament size evolution will be explained later. We further correlate the gel status with the zeta potential and the DLS data (Fig. 2, B and C, and figs. S5 and S6). The absolute value of the zeta potential increased, and the maximum *d*_h_ decreased following the black gel, black powder, brown gel, and brown powder, suggesting increased solution stability and decreased network development, in line with the analysis discussed before. In addition, we characterized the salting-out effectiveness by the low-threshold gelation concentration (*c*_s_) of salts and proved it to follow the Hofmeister series (Fig. 2D and fig. S7). All experiments above suggest that both the form (gel to powder) and color (black to brown) variation trends are strongly correlated to the salting-out effect of specific cations dictated by the Hofmeister series.

Aside from the salting-out function, cations also play a role in removing ligands. It is found that weakly bound citrates leave the NPs during gelation, resulting in negligible residues in the final NP-fused 3D networks (fig. S8). This phenomenon can only be caused by the introduction of salts since other conditions remained unchanged. Considering the opposite charges between cations and citrates, one explanation might be that cations strip citrates away from NPs to allow the development of gel networks. For verification, we performed density functional theory (DFT) calculations focusing on the citrate-cation interaction, so as to compare stripping ability of different cations (see the Supplementary Materials). Binding energies (*E*_b_) are obtained by calculating the energy change of the following reaction

$${\text{RCOO}}^{-}\;+\;X^{+}\;\to \;\text{RCOOX}$$ (1)

where RCOO^−^ and X^+^ denote citrate and cation, respectively. We considered the solvent effects using the conductor-like screening model detailed in the Supplementary Materials. *E*_b_ represents the energy decrease of the cation-ligand binding process and reflects the citrate removal efficiency of the different cations. As shown in fig. S9, the binding energy of single-charged cations exactly followed the Hofmeister series of NH_4_^+^(0.44 eV) < K^+^(0.57 eV) < Na^+^(0.85 eV) < H^+^(6.59 eV). However, the order of H^+^ and double-charged cations following Ca^2+^(2.27 eV) < Mg^2+^(2.96 eV) < H^+^(6.59 eV), which was inversed in comparison to the Hofmeister series. Although the order of binding energies given by the calculation partially inversed compared to the Hofmeister series, it well conforms to the order of increasing charge density and suits the experimental phenomenon (Fig. 2A and table S1). Following the order of NH_4_^+^, K^+^, Na^+^, Ca^2+^, Mg^2+^, and H^+^, the as-prepared products roughly evolved from gels to powders and from black to brown regardless of anions. The plotting of *E*_b_ versus ligament sizes (fig. S9) further quantifies the trend of size evolution, generally displaying a positive relationship, which accords with the trend of color changes. It can be deduced that with increasing *E*_b_ from NH_4_^+^ to H^+^, citrates will be more efficiently removed and more active sites would be exposed. In this way, the isotropic growth from the enhanced van der Waals attraction between surfaces of gold NPs is promoted and overwhelms the anisotropic growth induced by anisotropic electrostatic repulsion, which might be responsible for larger ligament sizes (*30*). Meanwhile, for high charge density cations (e.g., Mg^2+^, Ca^2+^, and H^+^), the very strong cation-citrate interaction can induce the fast formation of aggregates. Usually, too fast reaction kinetics is not preferred for gelation because the unstable environment may retard the intermediates to develop intact interconnected networks. Whereas, by lowing the salt concentration from 33 to 3.3 mM (e.g., for MgCl_2_ or CaCl_2_), self-supporting gels can be derived, which is presumably attributed to the decelerated reaction kinetics. Hence, in general, we observe increased ligament size and unsupported products with higher *E*_b_.

In virtue of specific ion effects analyzed above, we control the ligament size from 9.4 nm for NH_4_SCN to 199.6 nm for HCl (Fig. 2E). Compared to the Hofmeister series, certain inversions (K^+^ versus Na^+^) in ligament sizes might be attributed to similarities of certain cations and to possible effects from anions. Statistics from time-lapse TEM imaging further revealed the growth mode of the NPs (Figs. 1G and 2F), where the network development and the ligament size increase occurred simultaneously. Combining the above results, we propose a possible gelation mechanism (Fig. 2G and fig. S10): (i) The original NPs instantly approach each other upon the addition of salts due to electrostatic screening; (ii) the ligands are partially stripped away from the NPs by opposite-charged cations; (iii) the NPs fuse together to form aggregates driven by the raised surface energy of uncapped NPs; (iv) the aggregates repeat the above process and grow along both the axial and radial directions; and (v) the gravity-driven settlement of aggregates facilitates the hydrogel formation at the bottom. During gelation, the cations not only can precipitate the NPs by salting-out effects but also can strip ligands away from the NPs by electrostatic attraction. These combined effects direct the cations to roughly follow the Hofmeister series, changing the products from gels to powders and from small to large ligament sizes. Further efforts, from both experimental and theoretical sides, to verify the as-proposed mechanism and reveal the reaction process will be made in the near future.

### Flexible manipulation of NMAs

**Fig. 3:**
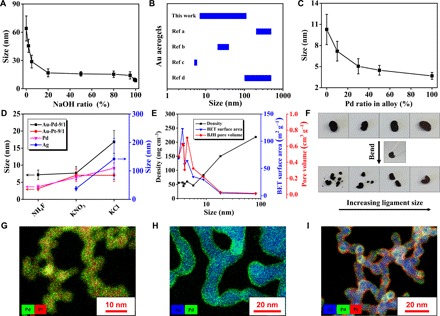
Versatile manipulation of NMAs. (**A**) Tailor the ligament size of gold gels by introducing NaOH/NaCl hybrid salts. (**B**) Ligament size of gold aerogels from different references [references (Ref) a to d corresponds to (*13*), (*9*), (*31*), and (*14*), respectively]. (**C**) The ligament size variation with Au/Pd ratio. (**D**) Ligament size modulation of Au-Pd, Au-Pt, Pd, and Ag gels using different salts. (**E**) The dependence of density, Brunauer-Emmett-Teller (BET) surface area, and Barrett–Joyner–Halenda (BJH) pore volume of aerogels versus ligament size. (**F**) Demonstration of the size-dependent mechanical properties of aerogels by bending with a tweezer. From left to right are Au-Ag-NH_4_F (5.8 ± 0.7 nm), Au-NH_4_SCN (8.9 ± 2.5 nm), Au-NH_4_NO_3_ (18.2 ± 4.0 nm), and Au-NaCl (64.0 ± 13.3 nm), respectively. (**G** to **I**) STEM–energy-dispersive x-ray spectroscopy (EDX) of three alloy gels with (G) homogeneous and (H and I) core-shell architectures.

The systematic manipulation of the ligament size and the corresponding physical properties of NMAs are the foundation for tailoring their specific applications, while it has not been realized previously. After a deep understanding of the gelation process and unlocking of specific ion effects, we devise the manipulation strategies in several manners. First, we deliberately selected specific salts (NH_4_SCN, NH_4_NO_3_, and KCl) as initiators based on the previous results (figs. S11 and S12), adjusting the ligament size from 8.9 ± 2.5 nm (for NH_4_SCN) to 80.3 ± 9.2 nm (for KCl) as characterized by TEM, scanning electron microscopy (SEM), and x-ray diffraction. In addition, we achieved an even larger ligament size of 113.7 ± 16.2 nm by MgCl_2_-induced gels. The color of KCl-induced aerogel is brown and quite close to that of bulk gold, while the other two aerogels with smaller ligament sizes appear black due to strong light absorption/scattering between nanosized domains (*29*). The change of the ligament size further tunes their density, specific surface area, and pore volume from 83.0 to 212.8 mg cm^−3^, 29.7 to 2.5 m^2^ g^−1^ (5843.4 to 492.5 m^2^ mol^−1^), and 0.218 to 0.010 cm^3^ g^−1^, respectively. Second, we used a double-salt system to continuously adjust the ligament size between the values given by the two single-salt systems. As shown in Fig. 3A, NaCl/NaOH hybrid salts quasi-continuously modulate the ligament size from 64.0 ± 13.3 to 8.9 ± 1.8 nm. Instead of a linear variation, the ligament size changed sharply by introducing a small amount of the second salt (2 to 10%) while keeping nearly unchanged in the broad middle region (NaOH% = 20 to 80%). Third, we modulated the ligament size by altering the concentrations of the used salts (NH_4_F was taken as example) or gold precursors, ranging from 8.0 ± 1.1 to 17.2 ± 4.6 nm and 6.9 ± 1.5 to 17.2 ± 3.9 nm, respectively (fig. S13). In virtue of the above strategies, the ligament sizes of the gold gels could be designed from 6.9 to 113.7 nm, covering a wide region not realized in previous reports (Fig. 3B) (*9*, *13*, *14*, *31*). Moreover, there is still ample space to extend the current systems and devise the gel parameters by elaborately selecting appropriate ions from the enormous ions library. We found that the used ligand, i.e., trisodium citrate, displayed—beyond its old role as only ligand or reductant—a new face as the gelation agent when its concentration is sufficiently high (fig. S14).

On the basis of the proposed gelation mechanism, the current system could be easily expanded to diverse noble metals (Ag, Pd, and Pt) and their alloys (fig. S15 and tables S2 and S3). Introducing a second metal to the gold system always leads to a reduction in ligament size, enabling fine-tuning the size from, e.g., 10.3 ± 2.1 to 3.7 ± 0.5 nm for the Au/Pd system (Fig. 3C and fig. S16). Further investigations showed that the Au, Ag, and Au/M (M = Pd, Pt) systems with higher Au content give relatively large ligament sizes and are easier to be modulated, while Pd and alloy systems with lower Au ratios resulted in small-sized gels (Fig. 3D and fig. S16). This phenomenon may partially result from the cohesive energy difference of metals, which positively correlates with their enthalpy of vaporization (Δ*H*_v_/kJ mol^−1^), following Ag(258) < Au(324) < Pd(362) < Pt(469) (*32*). A lower Δ*H*_v_ (Ag, Au) indicates weaker NP interactions, so that the NPs may need more coordinating neighbors to stabilize the structure, which leads to large ligament sizes (*33*), and vice versa. Summarizing the data from all prepared NMAs, we obtain ligament sizes from 3.1 to 142.0 nm, densities from 44.0 ± 3.6 to 212.8 ± 9.6 mg cm^−3^, Brunauer-Emmett-Teller (BET) surface areas from 122.7 to 2.5 m^2^ g^−1^ (15918.8 to 492.5 m^2^ mol^−1^), and Barrett–Joyner–Halenda (BJH) pore volumes from 0.704 to 0.013 cm^3^ g^−1^, demonstrating the superior manipulation capacity of the current strategy compared to reported methods to date (table S4). Correlating the ligament size with diverse parameters/properties showed that, generally, the density/mechanical bending strength increase with increasing ligament size, while the BET surface area/pore volume decrease (Fig. 3, E and F). This provides certain guidelines to engineer the physical parameters of NMAs. Notably, because of the weak mechanical strength, softness, and irregular shape of the as-prepared NMAs, quantitative characterization of their mechanical properties is still a great challenge and cannot be realized at this stage.

Among various NMAs, alloy aerogels always display a large application potential due to synergistic effects endowed by the multiple components, especially when deliberately designed secondary structures, e.g., hollow or core-shell architectures are included (*16*–*19*, *21*). As seen from energy-dispersive x-ray spectroscopy (EDX) analysis in the TEM, the spatial element distributions are either homogeneous [Pd-Pt (Fig. 3G)] or inhomogeneous [Au-Pd (fig. S17B) and Au-Pd-Pt (fig. S17D)] or show an imperfect core-shell structure [Au-Ag (fig. S17A)]. To control the spatial distribution of elements, we develop a straightforward dynamic shelling approach in the framework of the presented strategy. By simply introducing the second metal precursor during the initial gelation stage of the first metal, the second metal can nucleate and grow on the as-formed partially developed networks owing to the reduced energy cost by the heterogeneous nucleation process, eventually resulting in core-shell–structured gels. This approach not only can yield various bi- or trimetallic gels with well-defined core-shell structures (e.g., Au-Ag, Au-Pd, Au-Pt, and Au-Pd-Pt) but also can modulate the shell thickness (0.5 to 2.5 nm) by simply adjusting the ratios of the different components (Fig. 3, H and I, and fig. S18). In comparison with previously reported core-shell–structured NMAs fabricated either via the underpotential deposition followed by a galvanic replacement reaction (Pd*_x_*Au-Pt) (*21*) or via one-pot synthesis using a special reductant (PdPb-Pd) (*34*), the presented approach not only provides intact NMAs with well-defined and tunable core-shell structures but also manifests its considerable generality and simplicity owing to its straightforward mechanism.

### Properties and applications of NMAs

**Fig. 4:**
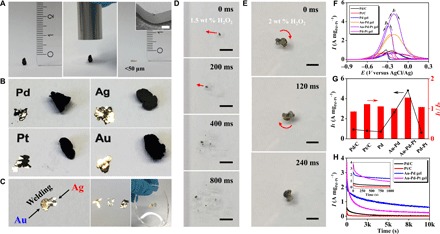
Plasticity and versatile applications of NMAs. (**A**) A piece of gold aerogel is manually pressed by ca. 98.5%. Scale bar, 30 μm (inset SEM image). (**B**) Original and compressed NMFs made of Au, Ag, Pd, and Pt. (**C**) Heterostructured welded Au-Ag aerogel by pressing, the logo “TUD” made by compressing Au, Ag, and Au-Pd-Pt alloy aerogels, and a gold TUD logo encased in PDMS. (**D** and **E**) Self-propelling behavior of (D) Ag aerogel and (E) compressed Au-Ag aerogel in H_2_O_2_ solution. wt. %, weight %. Scale bars, 5 mm. (**F** to **H**) Ethanol electro-oxidation performance, (F) cyclic voltammetry curves, (G) summarized *I*_f_ and *I*_f_/*I*_b_, and (H) chronoamperometry curves of different catalysts. (Photo credit: Ran Du.)

We study certain properties and potential applications of as-prepared NMAs to demonstrate their practical values. Most bulk metals appear lustrous and white. In contrast, nanostructured NMAs appear black, which has been explained to be due to light trapping in hierarchical microstructures because of multiple absorption and scattering by nearby grains (*29*). On the other hand, most metals exhibit remarkable ductility (i.e., the plasticity), which is explained by their strong dislocation emission ability (*35*). Therefore, it might be possible to induce a dark-to-shining transition by rearranging NMAs manually. As illustrated in Fig. 4 (A to C), figs. S19 and S20, and movie S2, various NMAs could be easily pressed from a height of millimeters to micrometers and thereby to regain metallic gloss via compacted nanostructured “mirror surfaces.” In addition, different aerogels could be welded together to form macroscopic heterostructures. Because of the extraordinary plasticity, NMAs could be arbitrarily shaped and encased in elastomers (e.g., polydimethylsiloxane) for potential use as flexible conductors.

Using catalytic oxygen evolution by decomposing H_2_O_2_ (*36*), we use a high–surface area silver aerogel to serve as a powerful self-propulsion device as an alternative to expensive Pt-based materials (Fig. 4D). The intensive catalytic reaction enables a maximum speed of 1.2 cm s^−1^ in 1.5 weight % H_2_O_2_ solution, comparable to that of Ag micromotors (>0.1 cm s^−1^) and bioelectrochemical self-propulsion devices (~1.0 cm s^−1^) (*36*, *37*). The motion form could be programmed using a heterostructured Au-Ag gel (Fig. 4E and movie S3). Upon reaction, it rotated automatically and displayed an angular speed of up to 168 rpm, which is due to its nonsymmetrical structure where only the Ag part can catalyze H_2_O_2_ for propulsion.

Last, we test the potential of NMAs in electrocatalysis by the alcohol electro-oxidation reactions. We performed cyclic voltammograms in the presence of ethanol, where the forward peak (i.e., the anodic peak) represents the oxidation of freshly adsorbed ethanol, while the backward peak indicates the removal of carbonaceous intermediates produced in the forward scan (*20*, *38*). Hence, the peak current density of the forward scan (*I*_f_) and the ratio of the peak current densities of forward/backward scans (*I*_f_/*I*_b_) can serve as indicators to evaluate the catalytic performance. As seen from Fig. 4, F and G, it is unexpected that high–surface area Pd and Pd-Pt aerogels (122.7 and 58.0 m^2^ g^−1^, respectively) show a lower performance compared to that of commercial Pd/C and Pt/C catalysts, presumably due to their less-continuous networks (fig. S15B). In contrast, Au-Pd and Au-Pd-Pt aerogels showed substantially higher performance with *I*_f_ of 2.65 and 4.82 A mg_Pd+Pt_^−1^, which are 2.8 to 6.1 times higher than that of commercial Pd/C or Pt/C catalysts and higher than most reported NMAs such as Pd-Cu, Pd-Ni, and Au-Ag-Pd aerogels (ca. 2.0 to 5.6 times compared to Pd/C) (*16*, *18*, *20*, *38*). Moreover, an *I*_f_/*I*_b_ of 1.37 is achieved for Au-Pd-Pt aerogels, displaying advantage over that of commercial (0.90 to 1.15) and most reported NMAs catalysts (0.90 to 1.28) (*16*, *18*, *20*). However, we observed the considerably current decay of Au-Pd and Au-Pd-Pt aerogels during a long-term test (Fig. 4H and fig. S21), which is a common issue for either commercial catalysts or reported Pd-based catalysts (*16*, *18*, *20*, *38*). In addition, Au-Pd and Au-Pd-Pt aerogels delivered superior electrocatalytic performance for the methanol oxidation reaction (see fig. S21), displaying high *I*_f_ of 1.22 and 2.06 mg_Pd+Pt_^−1^ compared to that of Pd/C and Pt/C (<0.5 mg_Pd+Pt_^−1^). This capability may enable the possible use of aerogels as anodic catalysts for various fuel cells. Aside from abundant active sites provided by the large specific surface area, the exceptional performance of NMAs may also rely on the intact and highly conductive 3D networks provided by the gold component, which can enhance the electrical conductivity, and thus facilitating efficient electron transfer in electrocatalysis.

To sum up, we have developed a specific ion–directed gelation strategy to rapidly fabricate and flexibly manipulate NMAs at room temperature from their NP solution. By activating specific ion effects and subtly regulating the NP-ion interactions, diverse single-/multi-NMAs with widely tunable compositions (Au, Ag, Pd, and Pt), ligament sizes (3.1 to 142.0 nm), specific surface areas (2.5 to 122.7 m^2^ g^−1^), and spatial element distribution (e.g., core-shell) are obtained. Combining experimental results and DFT calculations, the gel status is found to strongly depend on cation-ligand interactions, roughly following the Hofmeister series. On this basis, an overall picture of the sol-gel process is proposed, comprising electrostatic screening–induced aggregation, ligand stripping–directed NPs fusion, and gravity-driven sedimentation and gelation process. Last, several intriguing properties/applications of NMAs are demonstrated, including compression-induced dark-to-shining transition, devisable self-propulsion behavior, and high electrocatalytic activity toward ethanol/methanol oxidation. This study provides a conceptually new, general approach to fabrication of diverse NMAs, where flexible manipulations of chemical composition, ligament size, specific surface area, and spatial element distribution have been realized. In addition, certain perspectives in understanding the gelation process and underlying mechanism are proposed on the basis of experimental and theoretical results. Therefore, this work may pave the way for on-target designing versatile NMFs for various applications and studying the structure-performance relationship. Because of the enormous ion library and the generality of the gelation mechanism, the method we have introduced may also be adapted to versatile colloidal solution systems for on-demand manipulation for desirable applications.

## MATERIALS AND METHODS

### Fabrication of NMHs and NMAs

Hydrogels were synthesized by the two-step method at ambient temperature (~293 K), i.e., the preparation of NP solutions and the following gelation. As an example, the aqueous solution of trisodium citrate dehydrate (400.0 mM, 25.0 μl), HAuCl_4_·3H_2_O (32.5 mM, 30.8 μl), and NaBH_4_ (200.0 mM, 20.0 μl) were added successively to 4.93 ml of water and stirred for 30 to 60 min. After aging for ~1 day, NH_4_F (1.0 M, 555.0 μl) solution was added, and the mixture was subsequently grounded for 4 to 12 hours to acquire the hydrogel. After washing with water and exchanging with *tert*-butanol, the corresponding aerogel was obtained by freeze-drying for ~24 hours.

### Electrochemical measurements

All electrochemical tests were performed with a three-electrode system. The catalyst ink was prepared by dispersing a specific amount of catalyst in 2-propanol by sonication, and the accurate concentration of active components (Pd and Pt) was determined by inductively coupled plasma optical emission spectrometry. For electrocatalysis of ethanol and methanol oxidation, the loading of active components was ~20 μg cm^−2^, and electrochemical tests were performed in N_2_-saturated 1.0 M KOH aqueous solution containing 1 M ethanol or methanol.

## Supplementary Material

http://advances.sciencemag.org/cgi/content/full/5/5/eaaw4590/DC1

Download PDF

Movie S1

Movie S2

Movie S3

## SUPPLEMENTARY MATERIALS

Supplementary material for this article is available at http://advances.sciencemag.org/cgi/content/full/5/5/eaaw4590/DC1 Supplementary Materials and Methods Fig. S1. Characterizations of NP precursors. Fig. S2. Self-healing behavior of NH_4_F-induced gold hydrogels. Fig. S3. Digital photos of gelation behavior of gold NPs induced by different salts. Fig. S4. Digital photos of gelation behavior of gold NPs induced by four typical salts. Fig. S5. Zeta potential of gold NP solution after addition of different salts. Fig. S6. Time-lapse hydrodynamic size evolution during gelation. Fig. S7. The low-threshold gelation concentration and ligament sizes versus anions. Fig. S8. Residual analysis of as-prepared gold aerogels. Fig. S9. Energies derived by DFT calculations. Fig. S10. Proposed nanoscale force analysis and gelation mechanism. Fig. S11. Demonstration of the ligament size manipulation of gold aerogels using specific salts. Fig. S12. Nitrogen adsorption tests of different gold aerogels. Fig. S13. The relation of ligament size and precursors concentration. Fig. S14. Digital photos of gold gels initiated by other salts. Fig. S15. Demonstration and characterizations of diverse NMAs. Fig. S16. Ligament size manipulation of NMAs. Fig. S17. Scanning TEM–EDX analysis of different alloy gels prepared by one-step method. Fig. S18. High-angle annular dark-field scanning transmission electron microscopy imaging and EDX analysis of core-shell structured alloy gels. Fig. S19. SEM images of uncompressed aerogels. Fig. S20. Cross-sectional SEM images of compressed aerogels. Fig. S21. Electrocatalytic performance of different commercial and gel catalysts. Table S1. Summary of the gelation behavior of gold induced by different salts. Table S2. Summary of nitrogen adsorption data and ligament sizes of as-prepared aerogels. Table S3. Elemental analysis of different alloy aerogels. Table S4. Comparison of parameters of NMFs in literature. Movie S1. Demonstration of as-prepared black gels, brown gels, and black powders. Movie S2. Demonstration of pressing original aerogels into shining materials. Movie S3. Demonstration of self-propelled rotation of compressed Au-Ag aerogel. References (*39*–*50*)
