# Supplementary material for: Specific ion effects directed noble metal aerogels: Versatile manipulation for electrocatalysis and beyond
Source: Sci Adv. 2019 May 24;5(5):eaaw4590. doi: 10.1126/sciadv.aaw4590 (PMC6534393; doi:10.1126/sciadv.aaw4590)
Supplement: http://advances.sciencemag.org/cgi/content/full/5/5/eaaw4590/DC1 [file supp_5_5_eaaw4590__index.html]

Science Advances | Science Advances

## Supplementary Materials

**The PDF file includes:**

- Supplementary Materials and Methods
- Fig. S1. Characterizations of NP precursors.
- Fig. S2. Self-healing behavior of NH4F-induced gold hydrogels.
- Fig. S3. Digital photos of gelation behavior of gold NPs induced by different salts.
- Fig. S4. Digital photos of gelation behavior of gold NPs induced by four typical salts.
- Fig. S5. Zeta potential of gold NP solution after addition of different salts.
- Fig. S6. Time-lapse hydrodynamic size evolution during gelation.
- Fig. S7. The low-threshold gelation concentration and ligament sizes versus anions.
- Fig. S8. Residual analysis of as-prepared gold aerogels.
- Fig. S9. Energies derived by DFT calculations.
- Fig. S10. Proposed nanoscale force analysis and gelation mechanism.
- Fig. S11. Demonstration of the ligament size manipulation of gold aerogels using specific salts.
- Fig. S12. Nitrogen adsorption tests of different gold aerogels.
- Fig. S13. The relation of ligament size and precursors concentration.
- Fig. S14. Digital photos of gold gels initiated by other salts.
- Fig. S15. Demonstration and characterizations of diverse NMAs.
- Fig. S16. Ligament size manipulation of NMAs.
- Fig. S17. Scanning TEM–EDX analysis of different alloy gels prepared by one-step method.
- Fig. S18. High-angle annular dark-field scanning transmission electron microscopy imaging and EDX analysis of core-shell structured alloy gels.
- Fig. S19. SEM images of uncompressed aerogels.
- Fig. S20. Cross-sectional SEM images of compressed aerogels.
- Fig. S21. Electrocatalytic performance of different commercial and gel catalysts.
- Table S1. Summary of the gelation behavior of gold induced by different salts.
- Table S2. Summary of nitrogen adsorption data and ligament sizes of as-prepared aerogels.
- Table S3. Elemental analysis of different alloy aerogels.
- Table S4. Comparison of parameters of NMFs in literature.
- References (*39*–*50*)

Download PDF

**Other Supplementary Material for this manuscript includes the following:**

- Movie S1 (.mp4 format). Demonstration of as-prepared black gels, brown gels, and black powders.
- Movie S2 (.mp4 format). Demonstration of pressing original aerogels into shining materials.
- Movie S3 (.mp4 format). Demonstration of self-propelled rotation of compressed Au-Ag aerogel.

**Files in this Data Supplement:**

- Adobe PDF - aaw4590\_SM.pdf
