# Supplementary material for: Specific ion effects directed noble metal aerogels: Versatile manipulation for electrocatalysis and beyond
Source: Sci Adv. 2019 May 24;5(5):eaaw4590. doi: 10.1126/sciadv.aaw4590 (PMC6534393; doi:10.1126/sciadv.aaw4590)
Supplement: Download PDF [file aaw4590_SM.pdf]

Science Advances
AAAS

advances.sciencemag.org/cgi/content/full/5/5/eaaw4590/DC1

Supplementary Materials for

Specific ion effects directed noble metal aerogels: Versatile manipulation for electrocatalysis and beyond

Ran Du, Yue Hu*, René Hübner, Jan-Ole Joswig, Xuelin Fan, Kristian Schneider, Alexander Eychmüller*

*Corresponding author. Email: alexander.eychmueller@chemie.tu-dresden.de (A.E.); yuehu@wzu.edu.cn (Y.H.)

Published 24 May 2019, *Sci. Adv.* **5**, eaaw4590 (2019)
DOI: 10.1126/sciadv.aaw4590

The PDF file includes:

Supplementary Materials and Methods
Fig. S1. Characterizations of NP precursors.
Fig. S2. Self-healing behavior of NH_4F-induced gold hydrogels.
Fig. S3. Digital photos of gelation behavior of gold NPs induced by different salts.
Fig. S4. Digital photos of gelation behavior of gold NPs induced by four typical salts.
Fig. S5. Zeta potential of gold NP solution after addition of different salts.
Fig. S6. Time-lapse hydrodynamic size evolution during gelation.
Fig. S7. The low-threshold gelation concentration and ligament sizes versus anions.
Fig. S8. Residual analysis of as-prepared gold aerogels.
Fig. S9. Energies derived by DFT calculations.
Fig. S10. Proposed nanoscale force analysis and gelation mechanism.
Fig. S11. Demonstration of the ligament size manipulation of gold aerogels using specific salts.
Fig. S12. Nitrogen adsorption tests of different gold aerogels.
Fig. S13. The relation of ligament size and precursors concentration.
Fig. S14. Digital photos of gold gels initiated by other salts.
Fig. S15. Demonstration and characterizations of diverse NMAs.
Fig. S16. Ligament size manipulation of NMAs.
Fig. S17. Scanning TEM–EDX analysis of different alloy gels prepared by one-step method.
Fig. S18. High-angle annular dark-field scanning transmission electron microscopy imaging and EDX analysis of core-shell structured alloy gels.
Fig. S19. SEM images of uncompressed aerogels.
Fig. S20. Cross-sectional SEM images of compressed aerogels.
Fig. S21. Electrocatalytic performance of different commercial and gel catalysts.
Table S1. Summary of the gelation behavior of gold induced by different salts.
Table S2. Summary of nitrogen adsorption data and ligament sizes of as-prepared aerogels.
Table S3. Elemental analysis of different alloy aerogels.
Table S4. Comparison of parameters of NMFs in literature.

References (*39–50*)

Other Supplementary Material for this manuscript includes the following:

(available at advances.sciencemag.org/cgi/content/full/5/5/eaaw4590/DC1)

Movie S1 (.mp4 format). Demonstration of as-prepared black gels, brown gels, and black powders.
Movie S2 (.mp4 format). Demonstration of pressing original aerogels into shining materials.
Movie S3 (.mp4 format). Demonstration of self-propelled rotation of compressed Au-Ag aerogel.

Supplementary Materials and Methods

Experimental Procedures

Reagents and Materials

All reagents, such as hydrogen tetrachloroaurate (III) ($HAuCl_4 \cdot 3H_2O$), potassium tetrachloropalladate (II) (K_2PdCl_4), potassium tetrachloroplatinate (II) (K_2PtCl_4), trisodium citrate dihydrate, sodium borohydride ($NaBH_4$), and others were purchased from Sigma-Aldrich or Alfa-Aesar and used without further purification.

Fabrication of Single-Metal Noble Metal Hydrogels (NMHs)

Hydrogels were synthesized by a two-step method at ambient temperature (~293 K), dividing into nanoparticles (NPs) preparation and hydrogels formation. The fabrication process of a NH_4F-induced gold hydrogel is described as an example as follows:

Preparation of Gold Nanoparticles (NPs) Solution

Aqueous solution of trisodium citrate dehydrate (400 mM, 25 μL) and $HAuCl_4 \cdot 3H_2O$ (32.5 mM, 30.8 μL) were added successively in 4.93 mL water and stirred for ~15 min. Then freshly prepared $NaBH_4$ aqueous solution (200 mM, 20 μL) was rapidly injected, followed by stirring for 30~60 min. The molar ratio of metal salt (M), ligand (L), and reductant (R) is 1/10/4. The as-prepared NP solution (c_M=0.2 mM) was aged for ca. 1 day before hydrogel preparation. To prepare high-concentration gold NP solution ($c_M \geq 0.5$ mM), β-alanine was used as the ligand to replace the trisodium citrate.

Preparation of Gold Hydrogels

Aqueous solution of NH_4F (1 M, 555 μL) was added in as-prepared gold NP solution (5 mL), stirring for 10~20 s, followed by grounding 4~12 h to get free-standing gold hydrogel. The as-prepared gel is washed by a large amount water for 4~5 times with a total duration of 3 days to remove possible residues. For NP precursor solutions with different c_M, the final concentration of NH_4F (c_{salt}) was fixed to 100 mM. For hydrogels prepared by using different c_{salt}, the c_M was fixed to 0.2 mM.

For scale-up production, aqueous solution of trisodium citrate dehydrate (400 mM, 4 mL) and $HAuCl_4 \cdot 3H_2O$ (32.5 mM, 4.92 mL) were added successively in 788 mL water and stirred for ~15 min. Then freshly prepared $NaBH_4$ aqueous solution (200 mM, 3.2 mL) was rapidly injected in the above mixture, followed by stirring for 30~60 min. Afterwards, aqueous solution of NH_4F (1 M, 88.9 mL) was quickly added, stirring for ~20 s, followed by grounding ~12 h to allow complete reaction.

Fabrication of Alloy NMHs

Alloy hydrogels were synthesized by either one-pot or dynamic shelling approach (DSA). The latter strategy could be used to controlled fabricate core-shell structured gels.

For one-pot method, the procedure is the same to that of single-metal hydrogels, except that single metal precursor salts were replaced by two or more metal precursor salts. The molar ratio of different metal precursor salts is 1:1 for bi-metallic system and 1:1:1 for tri-metallic system, and the total concentration of metal salts in final solution is fixed to 0.2 mM.

For dynamic shelling approach, Au-Pd (molar ratio 1/1) system is taken as an example. Aqueous solution of trisodium citrate dehydrate (400 mM, 25 μL), $HAuCl_4 \cdot 3H_2O$ (32.5 mM, 15.4 μL), and

$NaBH_4$ aqueous solution (200 mM, 20 μL) were added in 4.93 mL water stepwise under stirring. After ~5 min, aqueous solution of NH_4F (1 M, 555 μL) was added and stirring was kept for ~10 s before switching off. After grounding for ~2 min, aqueous solution of K_2PdCl_4 (32.5 mM, 15.4 μL) was added under stirring for ~10 s. Thereafter, the mixture was grounded for ~12 h to acquire core-shell structured hydrogels.

Preparation of Noble Metal Aerogels

After purification by water, hydrogels were solvent-exchanged with tert-butanol for 2~3 times. Afterwards, wet gels were flash freezed by liquid nitrogen and remained at -196 ºC for ~10 min to enable complete freezing. The frozen samples were put into the chamber of freeze drier (TOPTI-12S-80) and dried for 12~24 h at ~1Pa. The temperature of the cold trap was set to -80 ºC.

Compression of Aerogels

To make a lustrous and solid aerogel pellet, the as-prepared aerogel was pressed manually by using a polished stainless-steel cylinder for a few seconds. To make the hetero-structured compressed Au-Ag aerogel, the original Au and Ag aerogel were placed together with a small overlapping fraction, followed by compressing as described above.

Computational Procedures

Macroscopic Force Analysis during Gelation Process

In solution, the aggregate is imposed by gravity (*G*), buoyancy (*f*), and viscous drag (*F*).*(39)* When the aggregate falls down, the direction of *F* is the same with that of *f*, while opposite with that of *G*. To facilitate estimation, given that all aggregates during gelation have the similar shape (quasi sphere) and density, only differ in sizes. *G*, *F*, *f*, and the volume of aggregate (*V*) could be expressed as follow

$$G=mg=\rho gV$$

$$f = \rho_w gV$$

$$F = 3\pi\eta dv$$

$$V = \pi d^3 / 6$$

Where ρ , ρ_w , η , *d*, and *v* denote the density of aggregate, density of solution, viscosity of solution, equivalent diameter of aggregate, and velocity of the aggregate.

For *F* that is expressed by Stock's law, the laminar flow need to be met, where the Reynolds number (Re) should be very small, e.g. less than 10 for the system of spheres. In our system, assume the viscosity and density of solution is close to the water (~10^{-3} Pa s and 10^3 kg m^{-3}), the maximum velocity (v_{max}) of the aggregate is 10 times of average settling down velocity (v_s), and the largest size of aggregate in solution is 100 μm. Since the precipitate appeared usually after 1 hours upon reaction for a system with a solution height of 5 cm, so the v_s should be less than 5 cm h^{-1}, thus a v_{max} of 50 cm h^{-1} was adopted. Then the up-limit of *Re* is estimated as follows and suggests a pure laminar flow condition

$$\mathrm{Re}=\frac{\rho d v_{\max}}{\mu}=\frac{1000*10^{-4}*(\frac{0.5}{3600})}{0.001}=0.0139$$

Then the total force F_{tot} could be expressed as

$$F_{tot} = mg - \rho_w gV - 3\pi\eta dv = mg(1-\rho_w/\rho) - 3\pi\eta v m^{1/3}(\frac{6}{\pi\rho})^{1/3}$$

Therefore, the acceleration (*a*) at certain velocity, and the maximum velocity (v_m) at the force equilibrium state (F_{tot}=0) are derived as

$$a = F_{tot} / m = -m^{-2/3} * 3\pi\eta v(\frac{6}{\pi\rho})^{1/3} + g(1-\rho_w/\rho)$$

$$v_m = \frac{gd^2(\rho\text{-}\rho_w)}{18\eta}$$

From above equation, it's clear that acceleration a is determined by the viscous drag term, which is positively related to the mass of aggregate. Hence, at the same velocity, a of big aggregate is always larger than that of the smaller one. So given that the initial velocity is zero, the velocity of larger aggregate should be always larger than that of the smaller one. At the final equilibrium state, the equilibrium speed is also positively related to the size, i.e. the mass of aggregate. Therefore, during whole gelation process, large aggregates would fall down all the way quicker than smaller ones. In this way, during gelation process, the gradually formed large aggregates will continuously fall down and eventually precipitate and gelate at the bottom of the vial.

Density-functional theory (DFT) Calculations

DFT calculations were carried out by using the Amsterdam Density Functional package.*(40-42)* The generalized gradient approximation (GGA) with the Perdew-Burke-Ernzerhof (PBE) functional*(43)* was employed together with a TZP basis set. The convergence criteria for energy and nuclear gradients were set to 10^{-3} Hartree and 10^{-3} Hartree/Å, respectively.

The initial structures were generated as follows: a high-temperature trajectory of a single citric-acid molecule was used to scan the conformational space with an approximate DFT method;*(44)* the optimized lowest-energy structure was taken as the initial structure and a single acidic proton was exchanged for the respective cations (Na^+, K^+, NH_4^+, Mg^{2+}, Ca^{2+}). All structures generated in this way were optimized with full DFT afterwards. In case of the alkaline earth metals, the molecule was positively charged. Dispersion effects were included by using the three parameter correction developed by Grimme et al.*(45)*

To mimic the experimental environment, the conductor-like screening model (COSMO) of solvation as implemented in ADF was used. It includes the respective molecule in a molecule-shaped cavity constructed by the atomic radii and surrounded by a dielectric medium (here: water). Taken single-charged cations as example, the binding energies (E_b) was calculated based on following equation

$$\mathrm{RCOO^-} + \mathrm{X^+} \rightarrow \mathrm{RCOOX} \qquad (1)$$

where $RCOO^-$ and X^+ denote deprotonated citrate and cation, respectively. E_b is defined $E_b = E_{RCOOX} - E_{RCOO-} - E_{X+}$ (we will display and discuss absolute values for convenience).

Characterizations

Microscopy Characterization

Scanning electron microscopy (SEM) analysis was performed on a Nova 200 NanoSEM scanning electron microscope. Samples were prepared by directly sticking on the conductive tape.

Transmission electron microscopy (TEM) analysis was carried out by using a FEI Tecnai G2 20 microscope operated at 200 kV. Samples were prepared by dispersing in acetone under ultrasonication (15 s to 120 s, depending on their dispersing ability), followed by dropping onto carbon-coated copper grids and drying at ambient temperature. For each formula, 2~3 TEM specimens from three different batches were analyzed and at least five different positions were surveyed for each one.

High-angle annular dark-field scanning transmission electron microscopy (HAADF-STEM) imaging and spectrum imaging based on energy-dispersive X-ray spectroscopy (EDX) were performed at 200 kV with a Talos F200X microscope equipped with an X-FEG electron source and a Super-X EDX detector system (FEI). Prior to STEM analysis, the specimen mounted in a high-visibility low-background holder was placed for 2 s into a Model 1020 Plasma Cleaner (Fischione) to remove contamination.

Optical imaging was acquired by Carl Zeiss Microscopy (Colibri 7), with the magnification of 63×10.

Diffraction Characterization

X-ray powder diffraction (XRD) was carried out in reflection mode on a Siemens D5000 X-ray diffractometer operated at a voltage of 30 kV and a current of 10 mA with Cu Kα radiation (λ = 1.5406 Å). The data were collected in the 20°–90° (2θ) range with a step size of $\Delta 2\theta = 0.02°$. The sample was fixed on the holder by Scotch tape. For single-metal system, the crystalline size could be estimated by the Scherrer equation applying crystallite-shape factor K=0.9.*(46)*

Spectroscopy Characterization

Fourier-transform infrared spectroscopy (FT-IR) spectra were recorded by Thermo Scientific Nicolet 8700 FT-IR Spectrometer configured with a Smart iTR diamond accessory.

Ultraviolet–visible spectroscopy (UV-vis) absorption spectroscopy was recorded on Cary 60 UV-Vis Spectrophotometer.

Thermal Properties Characterization

Thermogravimetric analysis (TGA) was conducted by Diamond TG-DTA/Spectrum GX with heating rate of 10 K min^{-1} under nitrogen atmosphere.

Element Analysis

X-Ray photoelectron spectroscopy (XPS) was performed by an Axis Ultra spectrometer (Kratos, UK) with a high-performance Al monochromatic source operated at 15 kV. The XPS spectra were taken after all binding energies were referenced to the C 1s neutral carbon peak at 284.8 eV, and the elemental compositions were determined from peak area ratios after correction for the sensitivity factor by CasaXPS.

Inductively coupled plasma optical emission spectroscopy (ICP-OES) was performed by Perkin-Elmer Optima 7000DV optical emission spectrometer.

Nitrogen Adsorption Measurement

Nitrogen adsorption experiments were performed with Quantachrome NOVA 3000e at 77 K. The sample was outgassed at 323 K for ~24 h under vacuum before measurement. The filling rod was used to reduce the dead volume and thus improving measurement accuracy. The specific surface area was calculated by using multi-point BET equation ($0.1<p/p_0<0.3$). The pore size distribution was derived by using the density functional theory method which is implanted in the software of the instrument. The total pore volume, which is calculated at $p/p_0=0.99$, in consistent with the value derived by BJH (Barret–Joyner–Halender) method.

Other Characterizations

Zeta potential and dynamic light scattering (DLS) tests were performed on ZETASIZER NANO (ZEN5600, Malvern Company). For both tests, the original solutions of gold nanoparticles and salts were purified separately with a 0.45 μm filter membrane before the tests. To reflect the real reaction conditions and to avoid the possible deviations incurred by dilution, the concentrations of all reactants (metal precursors, ligands, and salts) used here are exactly the same as described in the experimental part, i.e., 0.2 mM for metal precursors (~3.9 wt.% for gold), 2 mM for ligands, and 100 mM for salts. In this condition, although the absolute value of the hydrodynamic size derived from DLS might be partially affected due to the adopted high NP concentration (for DLS test, the typically used concentration is 0.1~1 wt.%), it is still meaningful to compare the time-lapse size evolution where the relative values count. The ligands and salts in solutions showed negligible effect on light (e.g. light transmission, absorption, scattering), which could be partially deduced from the fact that the solution is colorless and transparent with only salts and ligands. Therefore, their existence may not largely affect the accuracy of the results from the DLS tests.

Electrochemical Measurements

All electrochemical tests were performed with a three-electrode system on Autolab/PGSTAT 30 (Eco Chemie B. V. Utrecht, the Netherlands). Glassy carbon electrode (GCE, 3 mm in diameter), Ag/AgCl (saturated KCl aqueous solution) electrode, and platinum foil were used as working electrode, reference electrode, and counter electrode, respectively.

For modification of working electrode, ~1 mg catalyst was dispersed in 1 mL of 2-propanol by sonicating for ~30 min to acquire the catalyst ink. Then specific amount of ink was transferred on GCE electrode and evaporated at ambient temperature, followed by coating with 5 μL Nafion (0.5 wt % in ethanol). The concentration of Pd and Pt in ink was determined by ICP-OES, and the final loading of Pd and Pt (m_{Pd+Pt}) was calculated accordingly to be ~20 μg cm^{-2}. For commercial Pd/C (20 wt.% Pd on carbon black, Alfa) and Pt/C (20 wt.% Pt on carbon, Aldrich) catalyst, they were prepared in the same way, except that the initial concentration in 2-propanol was 4 mg mL^{-1}.

Cyclic voltammetry (CV) curves were conducted in nitrogen saturated 1 M KOH aqueous solution, with a voltage window between -1.0 and 0.5 V (vs. AgCl/Ag) and a scanning rate of 100 mV s^{-1}.

For electro-oxidation of ethanol, the test was performed under N_2 atmosphere in 1 M KOH aqueous solution containing 1 M ethanol. CV curves were recorded between -0.9 and 0.3 V (vs. AgCl/Ag) with a scanning rate of 50 mV s^{-1}. The peak current of the forward scanning (from negative to positive potential) and backward scanning are denoted as I_f and I_b, respectively. The stability test was conducted for 10000 s at the potential of forward peak current maximum. For electro-oxidation of methanol, 1 M ethanol was replaced by 1 M methanol, and all other conditions were remained the same.

Self-Propulsion Tests

For the self-propulsion test with original Ag aerogels, the solution is prepared by diluting 30 wt.% H_2O_2 aqueous solution with deionized water to 1.5 wt.% in a plastic Petri dish. Then a piece of Ag aerogel was carefully transferred in above solution and its behavior was recorded by video.

For the test with hetero-structured compressed Au-Ag aerogel, the sample was transferred in diluted H_2O_2 aqueous solution (2 wt.%), then the rotation behavior was observed and recorded.

The movement or rotation speed was determined by comparing the position of sample from different frames during certain period. The frame per second (FPS) of recorded video was 25, corresponding to the time resolution of 40 ms.

Figures

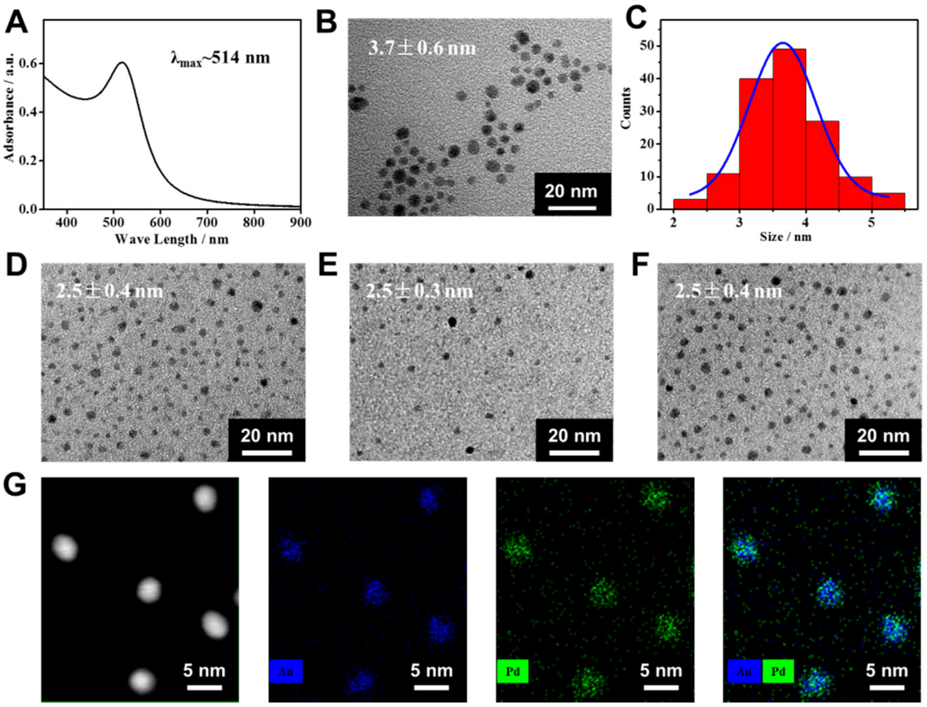

Fig. S1. Characterizations of NP precursors. (A-C) The UV-vis absorption spectrum, representative TEM images, and size distribution of gold NPs. (D-F) Representative TEM images of as-prepared silver, palladium, and platinum NPs. (G) HAADF-STEM image (left) and corresponding elemental distributions obtained by EDX analysis for Au-Pd alloy nanoparticle precursors (3.3±0.4 nm).

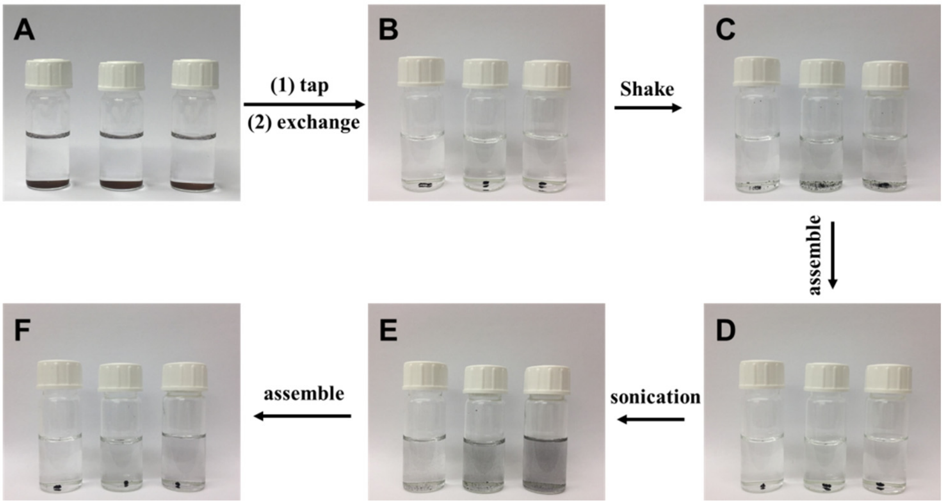

Fig. S2. Self-healing behavior of NH_4F-induced gold hydrogels. (A) Three identical gold hydrogels. (B) The solvent in middle and right vials are exchanged to pure water and acetone, respectively. (C-F) Three gels could readily self-healed to monolithic gels after destroying by either hand-shaking or sonication process. The self-healing process is achieved by tilting and tapping the vial, so as to bring gel pieces to contact each other for re-assembling into one piece. (Photo credit: Ran Du)

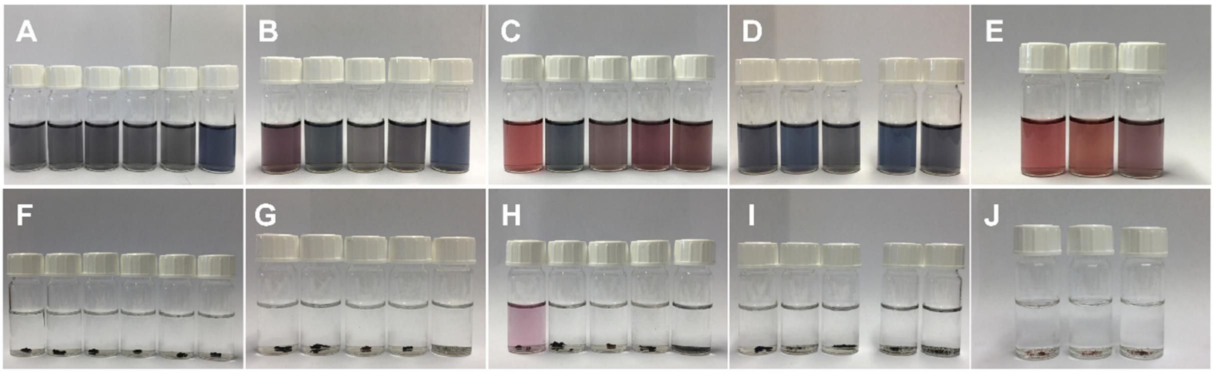

Fig. S3. Digital photos of gelation behavior of gold NPs induced by different salts. (A-E) After 5 min and (F-J) after 12 h. The employed salts from left to right are, (A,F) $(NH_4)_2SO_4$, NH_4F, $NH_3{\cdot}H_2O$, NH_4Cl, NH_4NO_3, NH_4SCN, (B,G) K_2SO_4, KOH, KCl, KNO_3, KSCN, (C, H) Na_2SO_4, NaOH, NaCl, $NaNO_3$, NaSCN, (D,I) $MgSO_4$, $MgCl_2$, $Mg(NO_3)_2$, $CaCl_2$, $Ca(NO_3)_2$, (E,J) H_2SO_4, HCl, HNO_3. It is of the same order as presented in fig. S6B. (Photo credit: Ran Du)

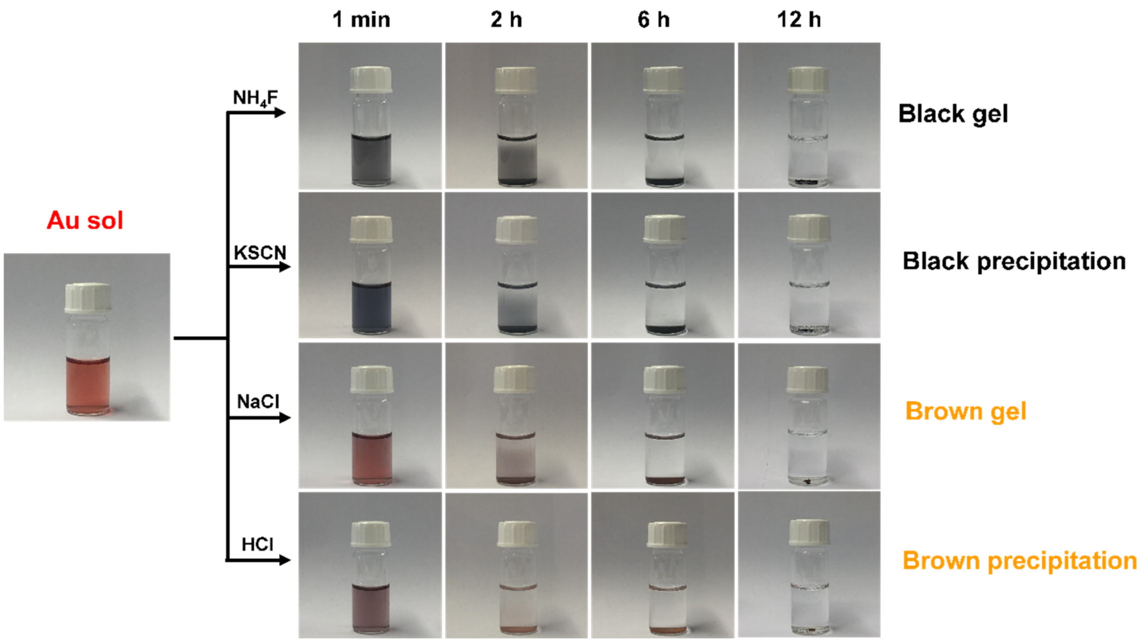

Fig. S4. Digital photos of gelation behavior of gold NPs induced by four typical salts. The black gel, black precipitation, brown gel, and brown precipitation are induced by NH4F, KSCN, NaCl, and HCl, respectively, representing four typical status of as-prepared hydrogels. (Photo credit: Ran Du)

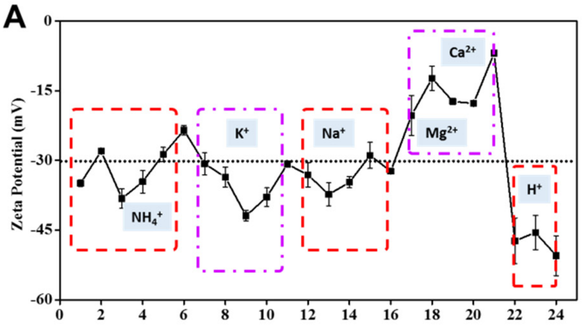

B

1	2	3	4	5	6
$(NH_4)_2SO_4$	NH_4F	$NH_3 \cdot H_2O$	NH_4Cl	NH_4NO_3	NH_4SCN
7	8	9	10	11	
K_2SO_4	KOH	KCl	KNO_3	KSCN	
12	13	14	15	16	
Na_2SO_4	NaOH	NaCl	$NaNO_3$	NaSCN	
17	18	19	20	21	
$MgSO_4$	$MgCl_2$	$Mg(NO_3)_2$	$CaCl_2$	$Ca(NO_3)_2$	
22	23	24			
H_2SO_4	HCl	HNO_3			

Fig. S5. Zeta potential of gold NP solution after addition of different salts. (A) Zeta potential of gold NP solution recorded 2 min after salt adding. (B) The corresponding salts in figure (A).

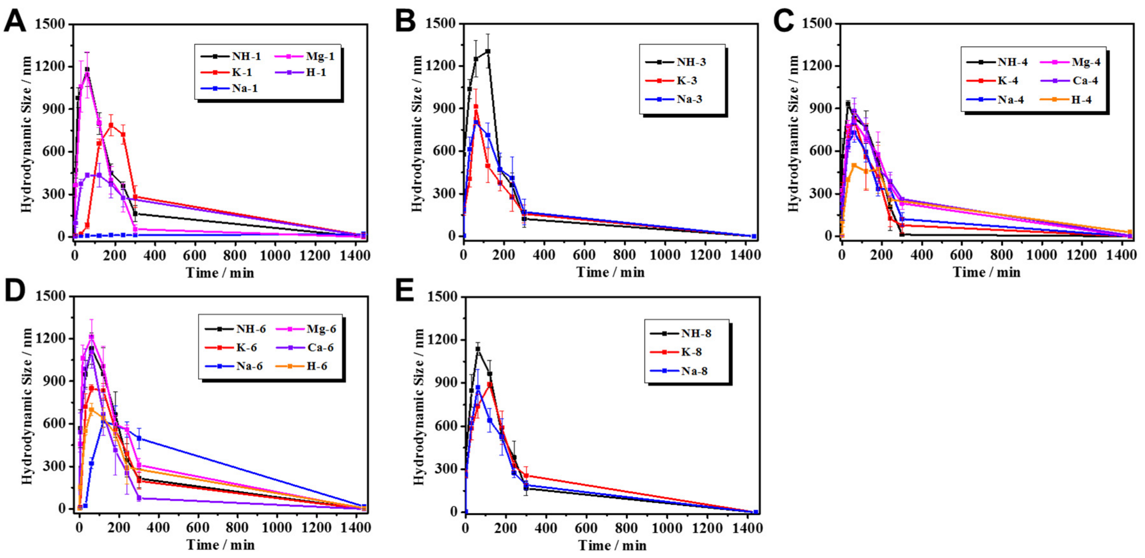

Fig. S6A. Time-lapse hydrodynamic size evolution during gelation, which are classified according to anions. (A-E) represent salts with anions of SO_4^{2-}, OH^-, Cl^-, NO_3^-, and SCN^-, respectively. In the legend, "NH" represent "NH_4^+", 1, 3, 4, 6, 8 represent SO_4^{2-}, OH^-, Cl^-, NO_3^-, and SCN^-, respectively.

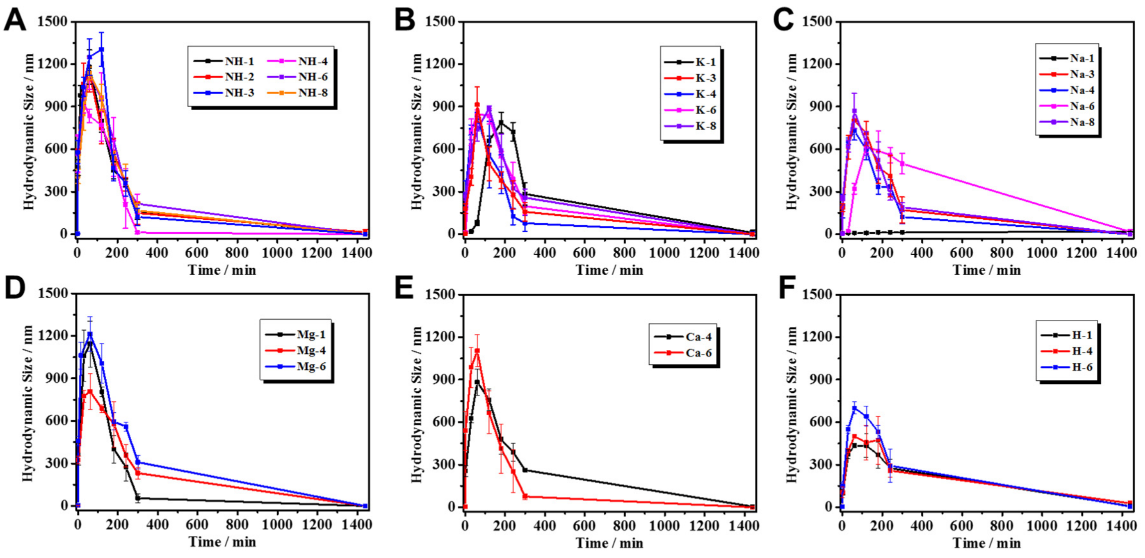

Fig. S6B. Time-lapse hydrodynamic size evolution during gelation, which are classified according to cations. (A-E) represent salts with cations of NH_4^+, K^+, Na^+, Mg^{2+}, Ca^{2+}, and H^+, respectively.

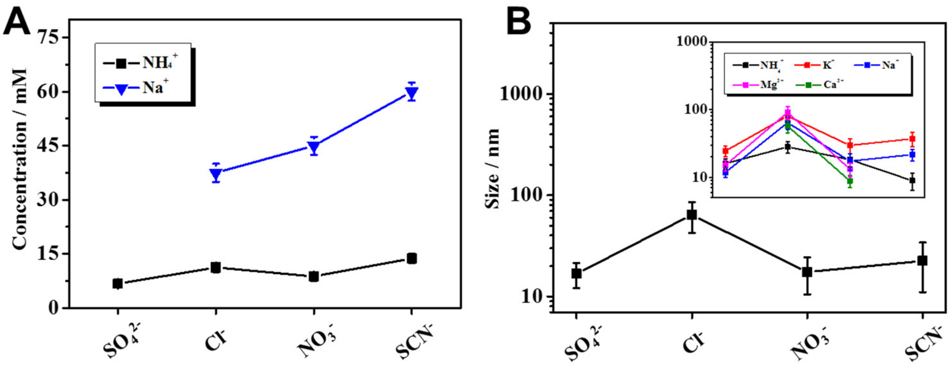

Fig. S7. The low-threshold gelation concentration and ligament sizes versus anions. The plotting of (A) low-threshold gelation concentration (c_{gel}) and (B) ligament size (averaged by employed cations in the inset diagram) of gel networks (d_g) against anions. In general, The c_{gel} increases roughly according to Hofmeister series by SO_4^{2-}, Cl^-, NO_3^-, and SCN^-; d_g achived the maximized value for Cl^-, while does not show obvious trend for other anions.

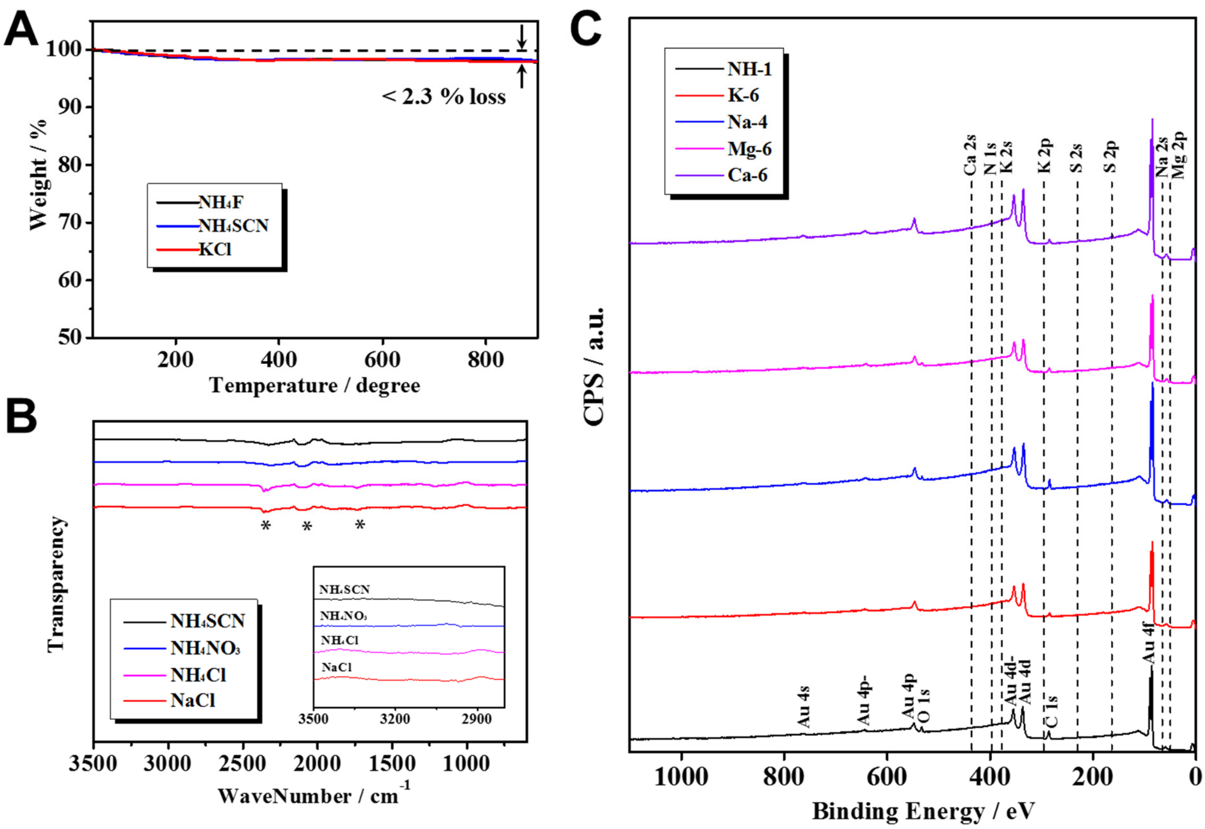

Fig. S8. Residual analysis of as-prepared gold aerogels. (A) TGA , (B) IR spectra, and (C) XPS spectra of gold aerogels directed by different salts. All results suggest few impurities (ligands, salts) exist in aerogels. The "*" in (B) denotes the peak from the noise of IR spectrometer.

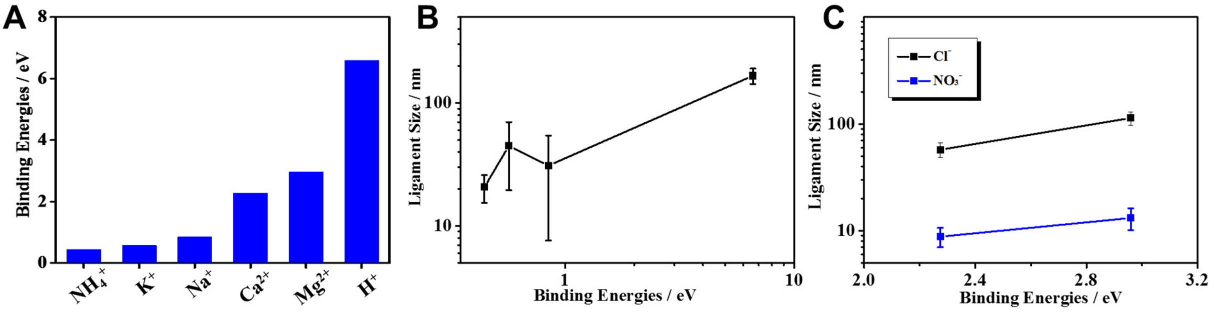

Fig. S9. Energies derived by DFT calculations. The plotting of the binding energies (E_b) versus (A) different cations, (B) the ligament sizes of single-charged ions (NH_4^+, K^+, Na^+, H^+), and (C) the ligament sizes of double-charged ions (Ca^{2+}, Mg^{2+}). The ligament sizes in B are obtained by averaging the sizes of products derived from single-charged cations paired with anions of SO_4^{2-}, Cl^-, and NO_3^-.

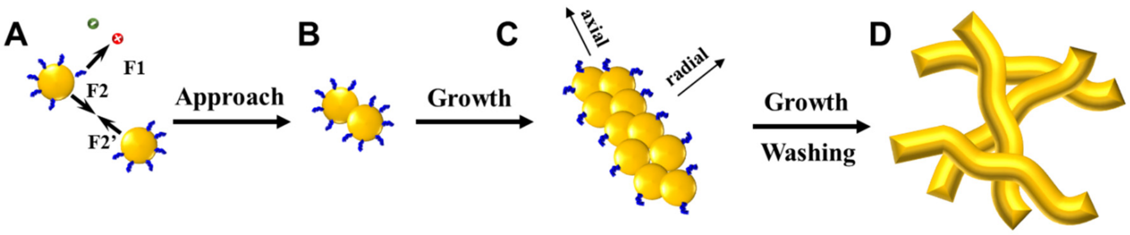

Fig. S10. Proposed nanoscale force analysis and gelation mechanism. (A) As two ligands-stabilized gold NPs approach induced by salts, the ligand on the nanoparticle is attracted by opposite-charged ions in solution (F1), thus leaving the bare surface of gold. Simultaneously, gold NPs attract each other driven by the raised surface energy of the uncapped gold surface, van der Waals attraction, and dipole-dipole interactions induced by the defects from removed ligands (F2, F2').*(30)* (B-D) NPs fuse together and grow along both axial- and radial direction depending on specific ions, finally evolving into 3D gel networks.

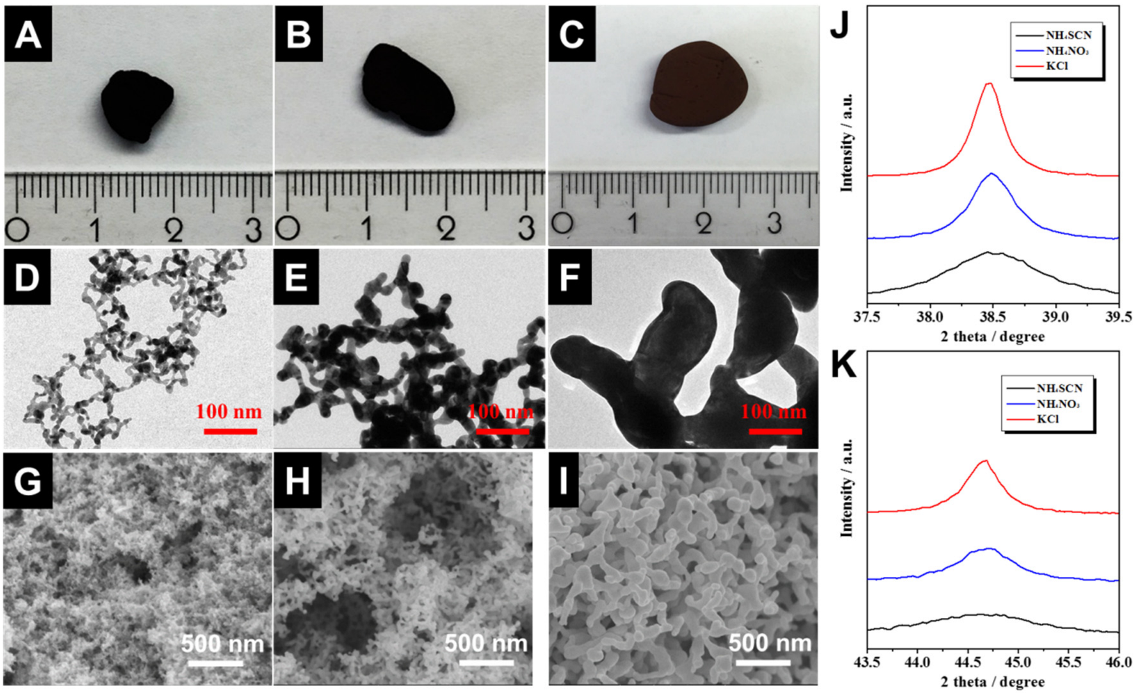

Fig. S11. Demonstration of the ligament size manipulation of gold aerogels using specific salts. The (A-C) digital photos, (D-F) representative TEM images, and (G-I) representative SEM images of gold aerogels prepared by NH_4SCN, NH_4NO_3, and KCl, respectively. (J-K) XRD patterns of Au (111) and Au (200) Bragg reflections of the corresponding gold aerogels. (Photo credit: Ran Du)

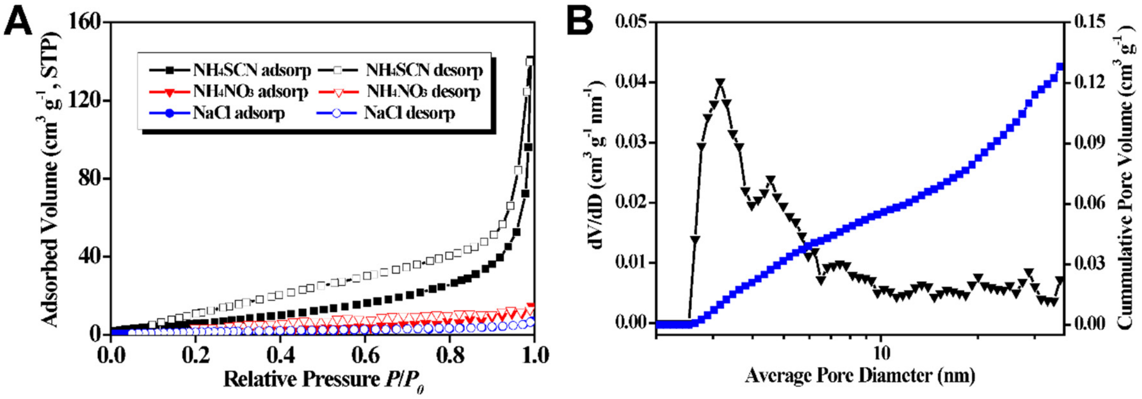

Fig. S12. Nitrogen adsorption tests of different gold aerogels. (A) Nitrogen gas adsorption curves of gold aerogels induced by NH_4SCN, NH_4NO_3, and NaCl. (B) Pore size distribution of NH_4SCN-induced aerogels was derived from the density functional theory as implemented in the instrument's software. The resulting average pore size is 3.2 nm for NH_4SCN-induced aerogels."

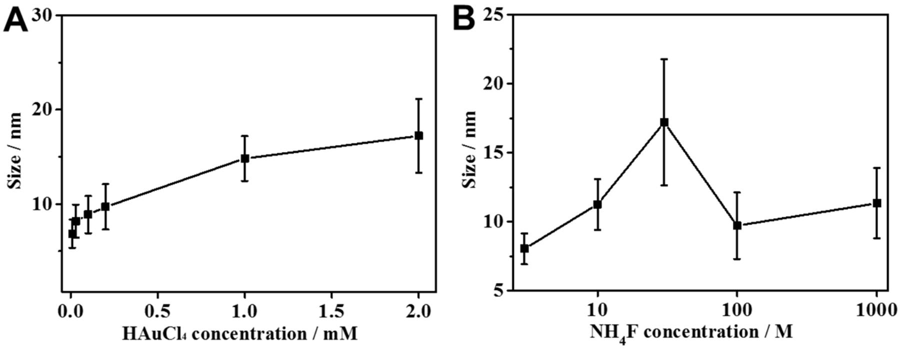

Fig. S13. The relation of ligament size and precursors concentration. The size of gold aerogels change with (A) gold precursor ($HAuCl_4$) concentration and (B) NH_4F concentration.

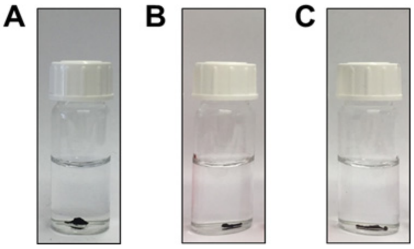

Fig. S14. Digital photos of gold gels initiated by other salts. Gold gels could be prepared by using (A) $NaClO_4$, (B) K_3PO_4, and (C) trisodium citrate (i.e. the ligand) as initiators. (Photo credit: Ran Du)

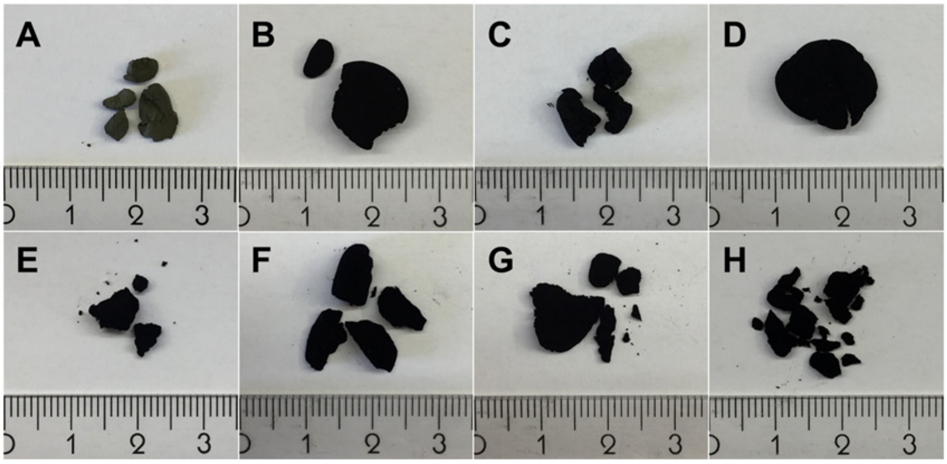

Fig. S15A. Digital photos of diverse noble metal aerogels. (A-C) Silver (Ag), palladium (Pd), and platinum (Pt) aerogels. (D-H) Alloy aerogels composed of Au-Ag, Au-Pd, Au-Pt, Pd-Pt, and Au-Pd-Pt, respectively. All aerogels are prepared by using NH_4F as the gelation initiator. (Photo credit: Ran Du)

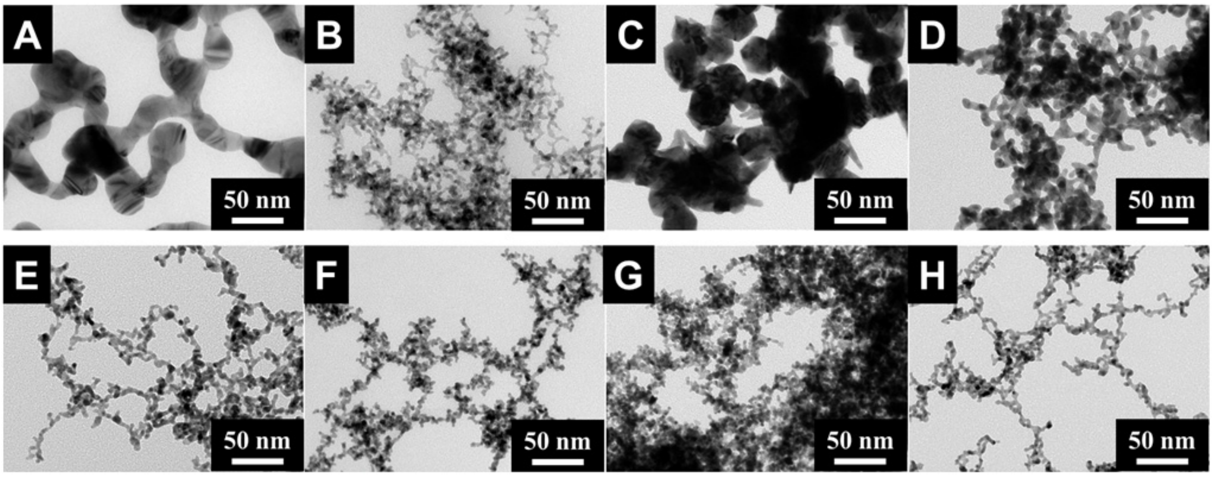

Fig. S15B. Representative bright-field TEM images of diverse noble metal aerogels. (A-C) Silver, palladium, and platinum aerogels. (D-H) Alloy aerogels composed of Au-Ag, Au-Pd, Au-Pt, Pd-Pt, and Au-Pd-Pt, respectively. (Photo credit: Ran Du)

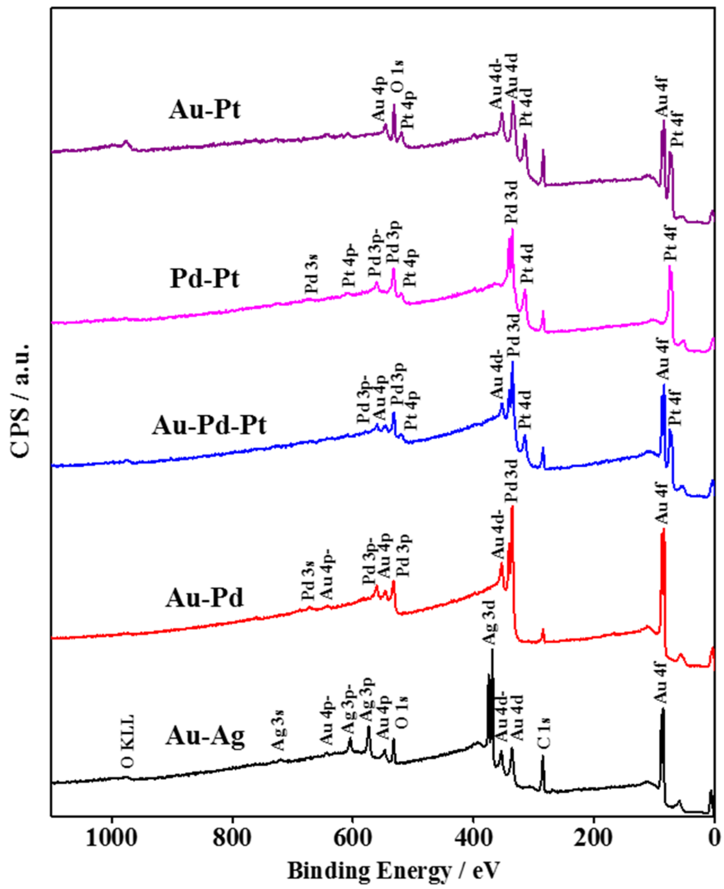

Fig. S15C. XPS spectra of diverse noble metal aerogels. (Photo credit: Ran Du)

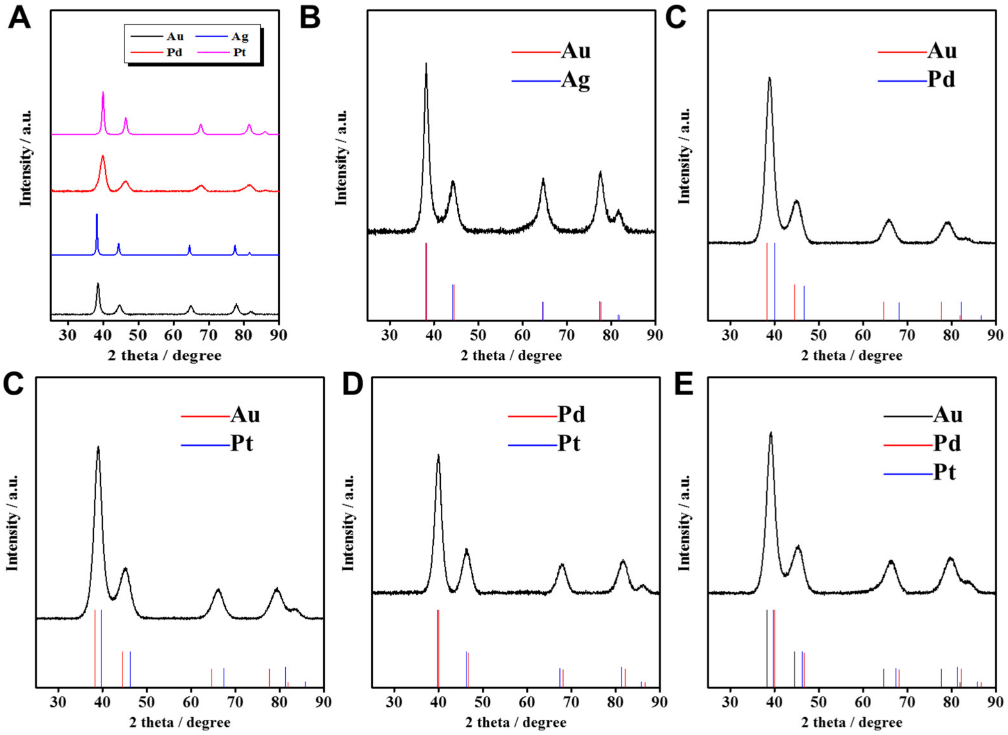

Fig. S15D. XRD characterization of noble metal aerogels. (A) Single noble metal aerogels. (B-E) Alloy noble metal aerogels composed by Au-Ag, Au-Pd, Au-Pt, Pd-Pt, and Au-Pd-Pt. (Photo credit: Ran Du)

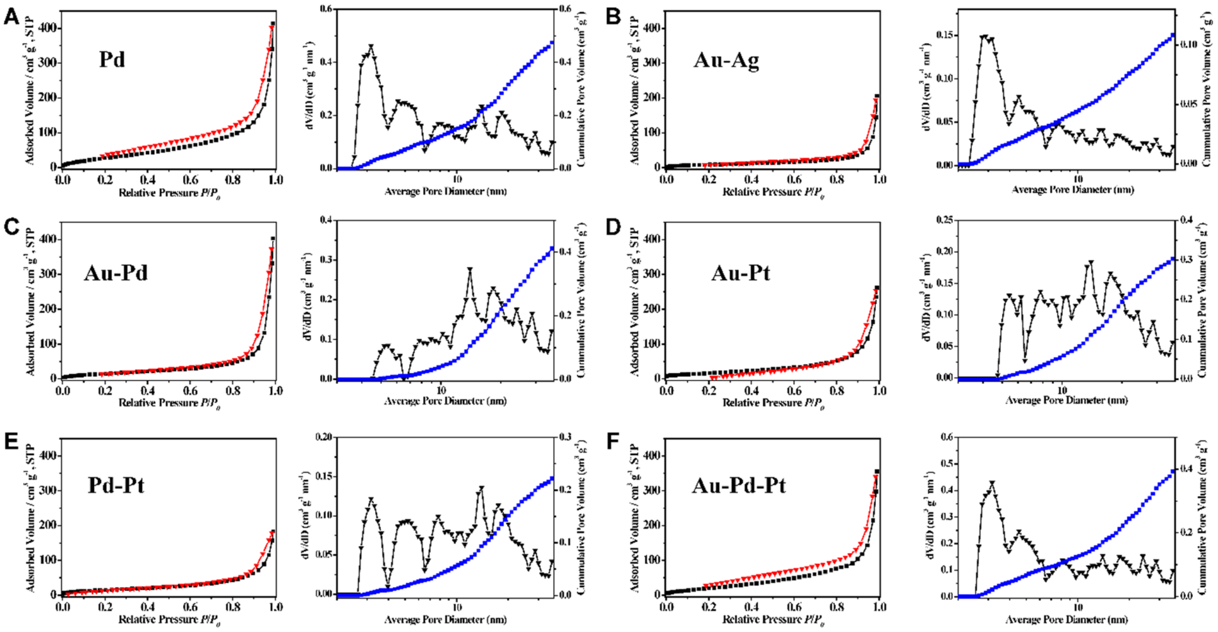

Fig. S15E. Nitrogen adsorption curves and pore size distribution (PSD) of noble metal aerogels. (A) Pd (3.2 nm), (B) Au-Ag (2.9 nm), (C) Au-Pd (14.0 nm), (D) Au-Pt (14.0 nm), (E) Pd-Pt (14.0 nm), and (F) Au-Pd-Pt (3.2 nm) aerogels. The PSD data was derived from the density functional theory as implemented in the instrument's software. The values in the brackets are the average pore sizes of the corresponding aerogels. (Photo credit: Ran Du)

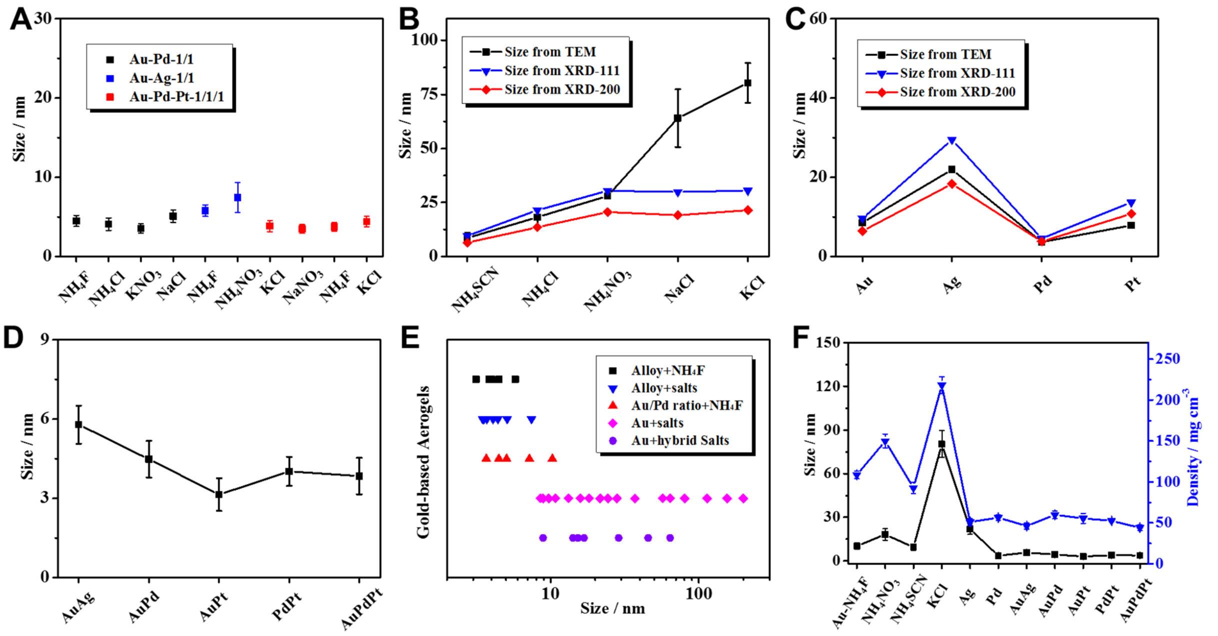

Fig. S16. Ligament size manipulation of NMAs. All sizes were determined from bright-field TEM images unless otherwise specified. Sizes of (A) gold-based alloy gels induced by different salts, (B) gold gels induced by different salts, (C) single noble metal gels induced by NH_4F, (D) alloy noble metal gels induced by NH_4F. (E) Summary of the size manipulation in gold-based gels by different strategies. (F) The correlation of sizes and densities of different noble metal aerogels.

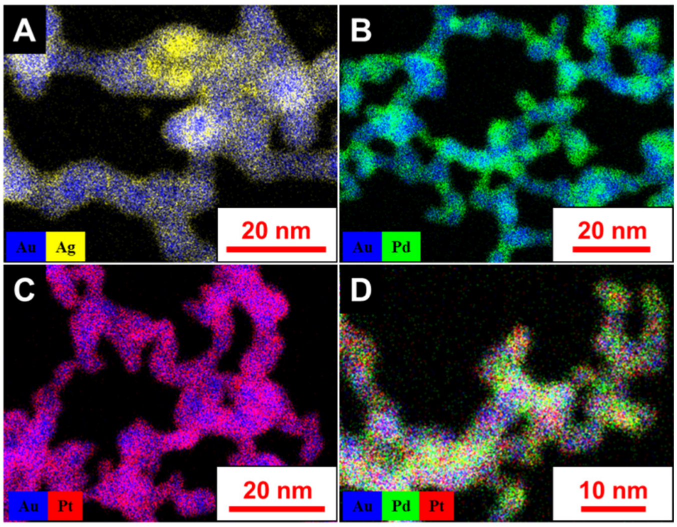

Fig. S17. Scanning TEM–EDX analysis of different alloy gels prepared by one-step method. (A) Au-Ag, (B) Au-Pd, (C) Au-Pt, and (D) Au-Pd-Pt gels were prepared by simultaneously reducing corresponding metal precursors in one pot with NH_4F as the initiator.

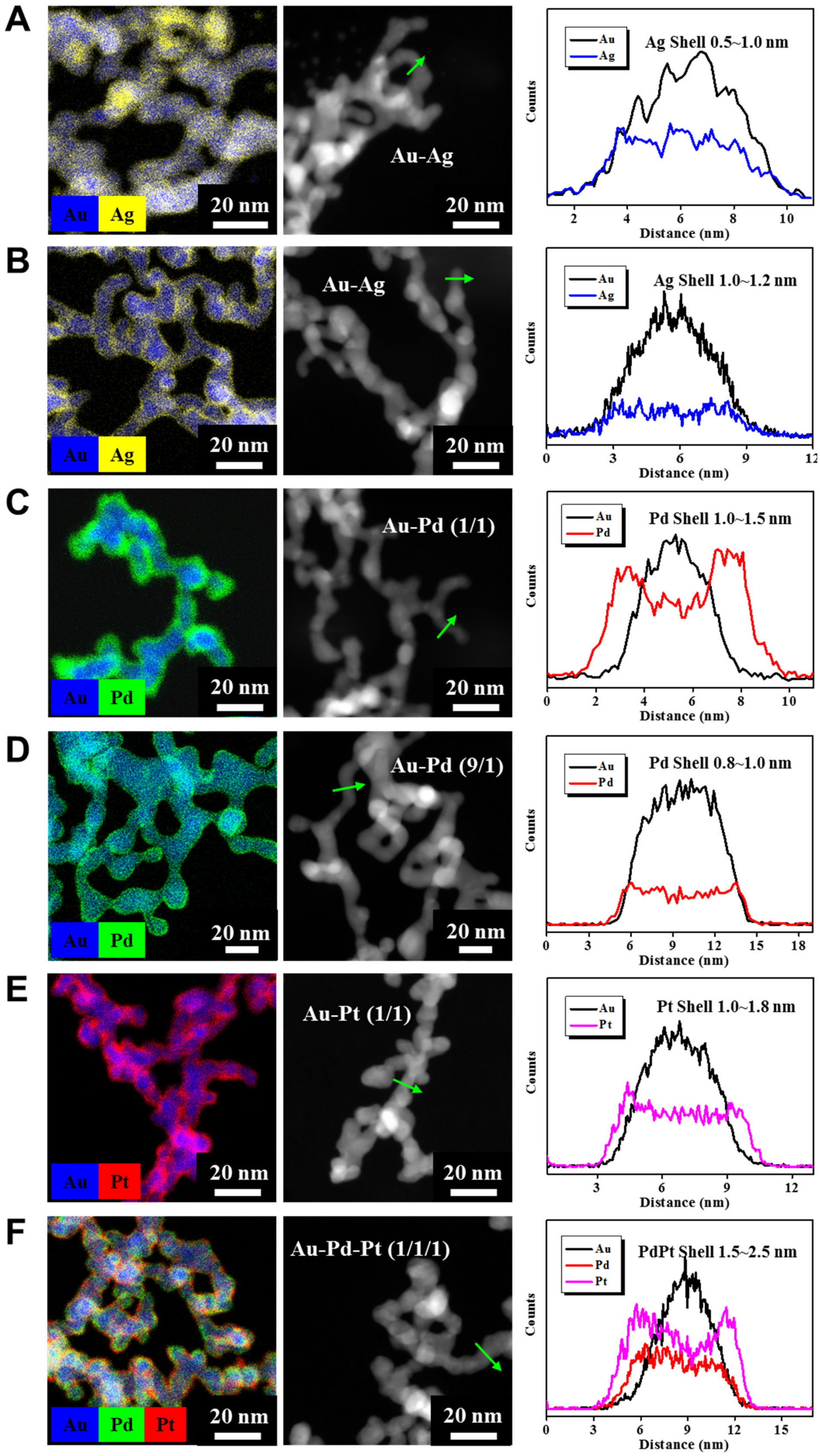
A
Au
Ag
20 nm
20 nm
Au-Ag
Au
Ag
Ag Shell 0.5~1.0 nm
Counts
Distance (nm)
B
Au
Ag
20 nm
Au-Ag
20 nm
Au
Ag
Ag Shell 1.0~1.2 nm
Counts
Distance (nm)
C
Au
Pd
20 nm
Au-Pd (1/1)
20 nm
Au
Pd
Pd Shell 1.0~1.5 nm
Counts
Distance (nm)
D
Au
Pd
20 nm
Au-Pd (9/1)
20 nm
Au
Pd
Pd Shell 0.8~1.0 nm
Counts
Distance (nm)
E
Au
Pt
20 nm
Au-Pt (1/1)
20 nm
Au
Pt
Pt Shell 1.0~1.8 nm
Counts
Distance (nm)
F
Au
Pd
Pt
20 nm
Au-Pd-Pt (1/1/1)
20 nm
Au
Pd
Pt
PdPt Shell 1.5~2.5 nm
Counts
Distance (nm)

Fig. S18. High-angle annular dark-field scanning transmission electron microscopy imaging and EDX analysis of core-shell structured alloy gels. (A) Au-Ag (1/1) gels prepared by one-pot method. (B-F) Au-Ag (1/1), Au-Pd (1/1), Au-Pd (9/1), Au-Pt (1/1), and Au-Pd-Pt (1/1/1) gels prepared by the dynamic shelling approach. The green arrows in the HAADF-STEM images indicate the line scanning direction.

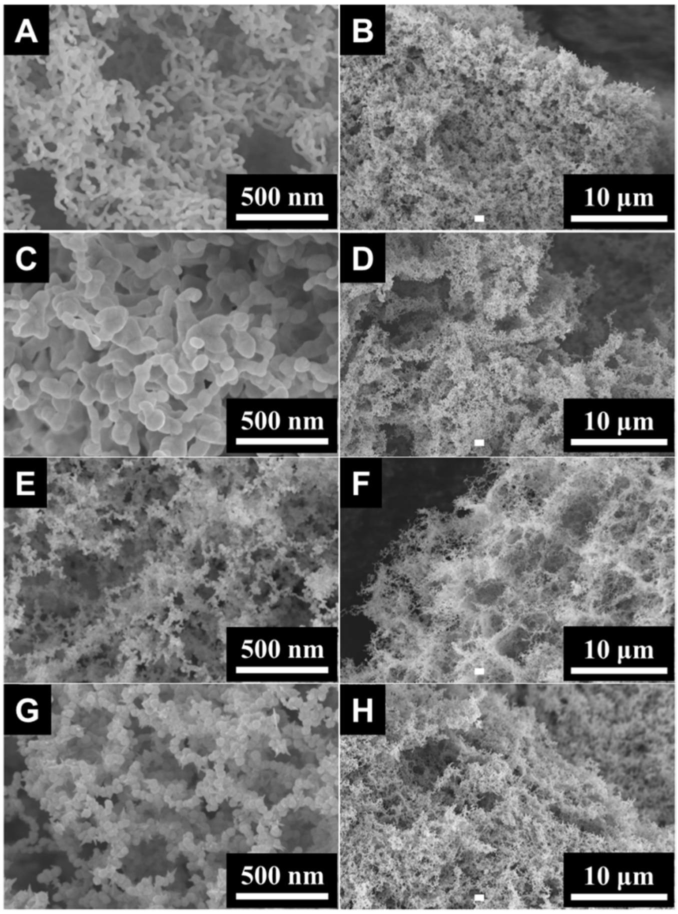

Fig. S19. SEM images of uncompressed aerogels. (A,B) Gold, (C,D) silver, (E,F) palladium, and (G,H) platinum gels induced by using NH_4F (for Au, Pd, Pt) or KOH (for Ag).

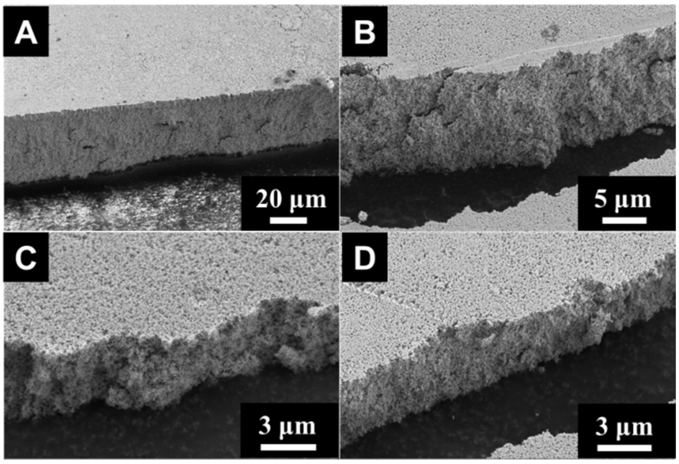

Fig. S20. Cross-sectional SEM images of compressed aerogels. (A) gold, (B) silver, (C) palladium, and (D) platinum.

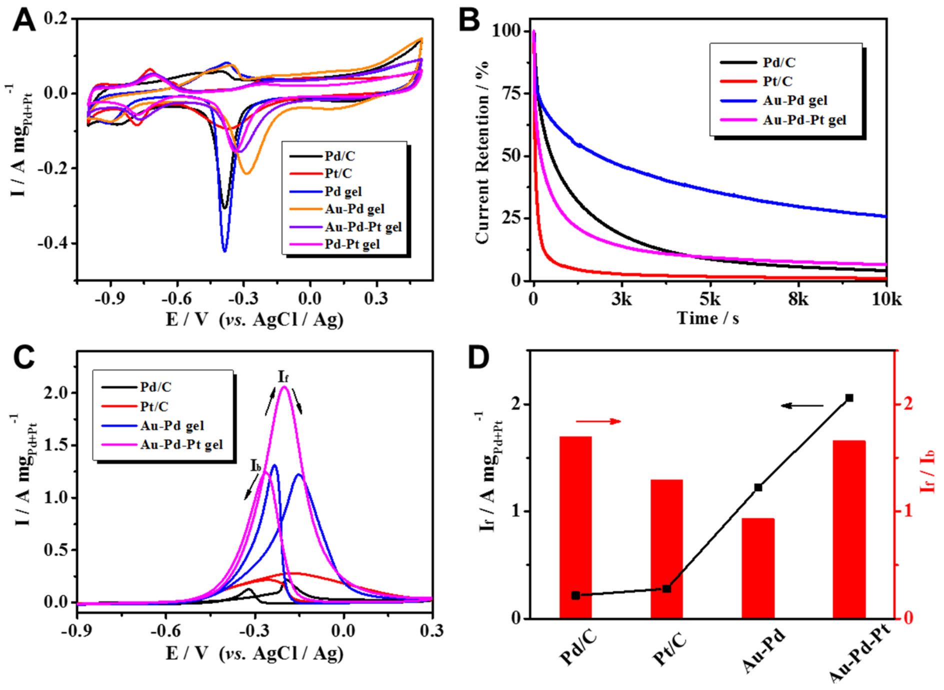

Fig. S21. Electrocatalytic performance of different commercial and gel catalysts. (A) CV curves in nitrogen-gas-saturated 1 M KOH solution, with a scan rate of 100 mV s^{-1}. (B) Current retention of different catalysts in 1 M KOH + 1 M ethanol solution. (C) CV curves of methanol oxidation catalytic reaction (MOR) conducted in 1 M KOH + 1 M methanol solution, with a scan rate of 50 mV s^{-1}. (D) Summarized I_f and I_f/I_b of different catalysts for MOR.

Tables

Table S1. Summary of the gelation behavior of gold induced by different salts. The salting-out ability decrease from SO_4^{2-} to SCN^-, and from NH_4^+ to Ca^{2+} according to Hofmeister series. The symbol of "√" and "×" indicate that the product is self-supporting gel or unsupported powders. The value in bracket is the relative surface charge density of cations and anions referred to NH_4^+. For calculation, the ionic radii were used according to literature *(47)*.

	**NH_4^+ (1.00)**	**K^+ (1.15)**	**Na^+ (2.11)**	**H^+ (11.11)**	**Mg^{2+} (8.45)**	**Ca^{2+} (4.38)**
**SO_4^{2-}**	√	√	√	×	√	
**F^-**	√					
**OH^-**	√	√	√			
**Cl^-**	√	√	√	×	×	×
**NO_3^-**	√	√	√	×	√	√
**SCN^-**	√	×	×			

Table S2. Summary of nitrogen adsorption data and ligament sizes of as-prepared aerogels. Specific surface area S_{BET} ($m^2\ g^{-1}$) was calculated in the partial pressure (p/p_0) range of 0.1-0.3. Total pore volume V_{tot} is derived at the volume at p/p_0=0.99. Average ligament size d_{ave} is derived from the statistic analysis of TEM measurements.

Entry	Metals	Salts	S_{BET} ($m^2\ g^{-1}$)	S_{BET} ($m^2\ mol^{-1}$)	V_{tot} ($cm^3\ g^{-1}$)	d_{ave} (nm)
1	Au	NH_4SCN	29.7	5843	0.218	8.9
2	Au	NH_4NO_3	4.7	933	0.023	18.2
3	Au	KCl	2.5	492	0.010	80.3
4	Au	$MgCl_2$	-	-	-	113.7
5	Au	$CaCl_2$	-	-	-	57.2
6	Au	HCl	-	-	-	199.6
7	Au	NH_4F	-	-	-	6.9~17.2[a]
8	Ag	NH_4F	5.2	563	0.030	21.9
9	Pd	NH4F	122.7	13055	0.021	3.7
10	Pt	NH4F	3.4	665	0.021	42.0
11	Au-Ag[b]	NH4F	34.8	6080	0.319	5.8
12	Au-Pd[b]	NH4F	63.9	9694	0.640	4.5
11	Au-Pt[b]	NH_4F	68.8	13488	0.405	3.1
12	Pd-Pt[b]	NH4F	58.0	8744	0.283	4.0
13	Au-Pd-Pt[c]	NH4F	95.8	15919	0.549	3.8

[a] The value represented the all available range of sizes by altering concentrations of $HAuCl_4$ and NH_4F (0.01~2 mM and 7.5~1000 mM, respectively).

[b] The molar ratio of the metals in original metal solution is 1/1.

[c] The molar ratio of the metals in original metal solution is 1/1/1.

Table S3. Elemental analysis of different alloy aerogels.

	Au-Ag	Au-Pd	Au-Pt	Pd-Pt	Au-Pd-Pt
**XPS**	1.00/1.14	1.00/1.01	1.00/1.04	1.00/1.06	1.00/0.98/0.94
**ICP-OES**	1.00/0.25	1.00/0.99	1.09/1	1.00/0.96	1.00/0.89/0.97

Table S4. Comparison of parameters of NMFs in literature.

Metals	Methods	Gelation Time	Size (nm)	Surface area (m^2 g^{-1})	Ref
Au	Conc[a] & H_2O_2	~1 week	100~500	Very small	14
Au	Dopamine	6~72 h	5~6	50.1	31
Au	Dealloy	/	20~40	3.7	9
Au	Conc[a] & FD[b]	/	200~500	Very small	13
Au	Salts	4~48 h	6.9~199.6	2.5~29.7	**This work**
Ag, Pt, Pd, Au-Ag, Au-Pd, Au-Pt, Pd-Pt, Au-Pd-Pt	Salts	4~12 h	3.1~142.0	5.2~122.7	**This work**
Au-Ag, Ag-Pt	Conc[a]	8~15 d	3~10	46~48	14
Au-Pd, Pd-Pt, Ag-Pd, Au-Ag-Pt	Conc[a] & 348 K	Several hours	3~6	57~79	48
Au-Ag-Pd	$(C(NO_2)_4$ & dealloying	16~24 h	3~5	76~269	38
Au-Ag, Pd-Ag, Pt-Ag	NaCl	1~2 d	/	32~42	25
MCu (M=Pd,Pt,Au)	343 K	6 h	5~6	20-40	16
Au-Pt	343 K	2~4 h	2~5	/	49
Ag	Dextran templating	/	~4000	0.1~0.5	10
Ag	$(C(NO_2)_4$	4~12 h	3~8	45-160	17
Pd	Conc[a] & Ca^{2+}	5 min ~ 2 month	3-10	40~108	15
Pd	β-CD	3~10 d	3~4	92	20
Pt	N_2H_4 in organic phase	1~5 h	3~8	30~40	50
Pd-Pt	$NaBH_4$	3~17 d	4~5	73~168	19
PdxAu-Pt	$NaBH_4$ & UPD[c] & GRR[d]	/	4~5	83~105	21

[a] Concentration of nanoparticles solution by centrifuge filters.

[b] Freeze drying.

[c] Underpotential deposition.

[d] Galvanic replacement reaction.
